# Beta Cell Proinsulin Response to Amino Acid Availability

**DOI:** 10.3390/cells15171584

**Published:** 2026-08-31

**Authors:** Yuting Ruan, Noah F. Gleason, Peter Arvan

**Affiliations:** Division of Metabolism, Endocrinology & Diabetes, University of Michigan, Ann Arbor, MI 48105, USAnoahglea@umich.edu (N.F.G.)

**Keywords:** amino-acids, translation, biosynthesis, pancreatic islet, diabetes

## Abstract

**Highlights:**

**What are the main findings?**

- At both normal and elevated glucose levels, pancreatic β-cells sense amino acid availability with increased phospho-eIF4E-BP1 and decreased phospho-eIF2α to stimulate proinsulin biosynthesis.
- Pancreatic β-cell nutrient selectivity includes importing certain amino acids (notably glutamine and serine) while exporting some others.

**What are the implications of the main findings?**

- In malnutrition-related (“type 5”) diabetes mellitus, blood glucose is abundantly available to pancreatic β-cells, but dietary proteins/amino acids are low.
- Even when glucose is available, amino acid insufficiency triggers diminished mTOR activity coupled with GCN2 activation that limits proinsulin (and insulin) biosynthesis.

**Abstract:**

Through fasting and feeding (both in vivo and in vitro), nutrient availability fluctuates over time. Pancreatic β-cells must respond to nutrients not only by regulated insulin secretion but also by insulin synthesis, which depends upon proinsulin. β-cells respond biosynthetically to glucose, but little is known about amino acids as regulators of proinsulin levels. We find that in INS1E (rat) β-cells, isolated murine islets, and isolated human islets—even in the presence of abundant glucose, proinsulin levels remain sensitive to reduced availability of other nutrients contained in β-cell/islet bathing medium. A low proinsulin level upon in vitro fasting is accompanied by diminished ribosomal phospho-S6 and phospho-eIF4E-BP1 plus increased phospho-eIF2α, reversed upon re-feeding. Even as extracellular glucose levels remained steady, INS1E cells consumed the majority of extracellular glutamine and serine (measured by mass spectrometry); concurrently, six nonessential amino acids (most notably proline and alanine, plus glutamic and aspartic acid) were actually exported from cells to media. Proinsulin suppression in response to amino acid limitation appears to be β-cell-autonomous, is linked to proinsulin biosynthesis, and is ameliorated upon GCN2 inhibition in INS1E cells. Upon re-feeding amino acid-containing medium, the mTORC1 inhibitor rapamycin fully suppressed phospho-S6 but did not block the recovery of proinsulin levels, whereas Torin1 inhibition of mTOR inhibited the amino acid-dependent increase in proinsulin biosynthesis. Altogether, these data indicate that inadequate amino acid-triggered β-cell signaling can blunt the proinsulin biosynthetic response, contributing to insulin deficiency—a finding that has potential relevance to the pathogenesis of malnutrition-related diabetes.

## 1. Introduction

High among the multiple tissue targets of genetic and acquired risk for type 2 diabetes (T2D) are pancreatic islets [1,2]—with defects resulting in impaired insulin secretion [3]. Numerous islet-related T2D risk alleles have been linked to β-cell insulin content, which reflects a balance between insulin biosynthesis (which is derived from proinsulin) and insulin secretion/turnover. Synthesis of proinsulin is essential for insulin production [4], which can become compromised in T2D and other forms of diabetes. While some T2D risk alleles, along with nutrient overload, may drive proinsulin biosynthesis in excess of the capacity of β-cells to fold the protein [5], other allelic variants and other environmental conditions may decrease proinsulin biosynthesis and thus predispose to insulin deficiency in this other way.

Proinsulin biosynthesis is acutely regulated by nutrients at the levels of translation, ER translocation, and protein folding. Some studies have simply equated nutrients needed for proinsulin biosynthesis with those that stimulate insulin release [6], with a primary focus on glucose [7]. However, nutrients stimulating synthesis and release are not identical—as just one example, arginine suppresses proinsulin biosynthesis while strongly stimulating insulin secretion [8,9]. Not only have the relationships of proinsulin synthesis to many physiological/pathophysiological nutrient exposures other than glucose remained unclear, but in recent decades, most studies of proinsulin biosynthesis have ignored amino acids by pulse-labeling pancreatic islets in buffered salt solutions [10,11,12,13,14,15,16,17,18], unlike classic older studies [19,20,21]. Mammalian cells require nine essential amino acids that cannot be synthesized de novo. There are few very unbiased experiments to establish whether β-cells show preference for consuming these nine or others (or perhaps all 20 amino acids) from the extracellular environment. β-cells can also receive amino acids via proteolytic degradation of intracellular or ingested proteins/peptides. We hypothesize that β-cell proinsulin synthesis exhibits a dependence on amino acid availability to a much greater extent than has previously been recognized.

Despite the paucity of research on amino acids as potential regulators of proinsulin synthesis, this phenomenon takes on considerable biological significance, as there is reason to believe that diminished amino acid availability to β-cells may contribute importantly to the disease now labeled as “type 5” (undernutrition-associated) diabetes mellitus [22,23]. This form of diabetes affects at least 25 million people globally and is particularly concentrated in Africa [24,25] and South Asia [26]—with a hallmark of insulin deficiency [27] as opposed to insulin resistance [28].

As part of an improved understanding of the nutritional dependence of insulin production, we published a preliminary study of the Min6 mouse pancreatic β-cell line, examining the impact of amino acid availability on proinsulin levels [29]. Here, we have sought to further validate the importance of amino acid availability for regulating proinsulin levels in pancreatic β-cells.

## 2. Materials and Methods

### 2.1. Reagents

All tissue culture reagents were from Invitrogen. SDS-PAGE 4–12% Bis-Tris or 12% Tris-Glycine NuPage gels were purchased from ThermoFisher (Waltham, MA, USA). MG132 was from Sigma-Aldrich (St. Louis, MO, USA); GCN2 inhibitor (HY-112654) and rapamycin (HY-10219) were obtained from MedChemExpress (Princeton, NJ, USA). Torin1 was obtained from Cell Signaling Technology (#14379, Danvers, MA, USA). TL-033 was obtained from Dr. T.W. Bell (University of Nevada, Reno, NV, USA).

### 2.2. Cell Culture and RPMI Feeding Protocol(s)

INS-1E rat pancreatic β-cells (originally obtained from Dr. C. Wollheim, U. Geneva, Switzerland) were cultured in RPMI-1640 medium supplemented with 10% fetal bovine serum (FBS), 10 mM HEPES, 1 mM sodium pyruvate, penicillin/streptomycin, and 0.05 mM β-mercaptoethanol. One independent experimental replicate corresponds to a separate cell culture performed on a different day; within an experiment, cells from a common pool were divided among the indicated conditions so that comparisons were matched within each experiment. For fasting/feeding experiments, cells were seeded into 12-well plates and cultured under a limiting-volume condition (90 μL medium per cm^2^ of surface area). Culture medium was replaced every 24 h. After 48 h of culture, cells were incubated with fresh complete RPMI-1640 medium containing either 5.5 mM or 11.1 mM glucose for 4 h, followed by an additional 5 h re-feeding period with media of varying nutrient composition prior to sample collection and analysis. For amino acid restriction experiments, RPMI-1640 medium containing 25% of the standard amino acid concentration [30,31] was prepared by diluting complete RPMI-1640 medium with 75% Hank’s balanced salt solution (HBSS), except in Supplemental Figure S7, in which RPMI lacking all amino acids was used as diluent. The final glucose concentration was maintained at either 5.5 mM or 11.1 mM, as indicated. Under all conditions, including 48 h of limited medium volume as well as 4 or 5 h after feeding diluted media, cell viability was confirmed to be maintained as determined by propidium iodide staining to detect cells with compromised membrane integrity (with formalin/methanol-fixed cells serving as a positive control). Puromycin labeling of cells is described in the legend to Supplemental Figure S4.

Total RNA was extracted from INS1E cells using the RNeasy Mini Kit (Qiagen, Hilden, Germany), and reverse-transcribed using the High-Capacity cDNA Reverse Transcription Kit (Applied Biosystems, Foster City, CA, USA). Quantitative real-time PCR was performed using SYBR Green qPCR Master Mix (Alkali Scientific, Fort Lauderdale, FL, USA) with the following primer sequences: *Ins1* forward 5′-CAATCATAGACCATCAGCAAGC-3′ and reverse 5′-CTGTTTGACAAAAGCCTGGG-3′; *Ins2* forward 5′-GAAGTGGAGGACCCACAAGT-3′ and reverse 5′-AGTGCCAAGGTCTGAAGGTC-3′; and *Actb* (β-actin, serving as the reference gene) forward 5′-CCCGCGAGTACAACCTTCT-3′ and reverse 5′-CGTCATCCATGGaCGAACT-3′. All qPCR reactions were performed in triplicate from 5 independent studies. Relative mRNA expression was calculated using the comparative Ct method and was compared between groups by one-way ANOVA with Tukey’s post hoc test.

### 2.3. Antibodies

Antibodies in this study include mouse mAb anti-rat proinsulin (CCI-17, RRID:AB_1107982, Novus, Littleton, CO, USA); mouse mAb anti-human proinsulin B-C junction sequence (Abmart, RRID: AB_2921300), rabbit mAb anti-HSP90 (RRID: AB_2233307), rabbit mAb anti-phospho-eIF2α (Ser51, RRID: AB_390740) and rabbit mAb anti-phospho-4E-BP1 (Thr37/46, RRID: AB_330966) from Cell Signaling, Danvers, MA, USA; mouse mAb anti-phospho-S6 ribosomal protein (Ser235) antibody (RRID: AB_2879523, Proteintech, Rosemont, IL, USA); mouse anti-puromycin (RRID:AB_2619605, DSHB, Iowa City, IA, USA); and mouse mAb anti-Vinculin antibody (RRID: AB_477617, Sigma-Aldrich, St. Louis, MO, USA).

### 2.4. Mouse Islet Isolation

Islets from 8 to 10 week-old C57BL/6J mice were isolated by collagenase digestion of the pancreas, followed by washing and density gradient centrifugation using Histopaque gradients (cat. #1119 and #1077, Sigma-Aldrich, St. Louis, MO, USA). Prior to each experiment, isolated islets were washed, handpicked, and incubated overnight at 37 °C in complete RPMI-1640 plus 10% FBS. Each experiment utilized islets prepared from two mice and pooled them before dividing into different treatment conditions. Islets were transferred to a limiting volume of fresh complete medium (100% RPMI-1640) or to amino acid-diluted medium (25% RPMI-1640 with glucose fixed at 11.1 mM). Islets were cultured for the indicated times before experimental analyses.

### 2.5. Human Islets

Five preparations of nondiabetic human pancreatic islets (≥85% purity, ≥85% viability, confirmed COVID-negative; insulin secretion data not available for each preparation) were generated at Prodo Laboratories (Aliso Viejo, CA, USA) or the Michigan Diabetes Research Center Islet Core facility:

Donor #1: 66-year-old female, BMI 32.4 (HbA1c 5.5%);

Donor #2: 62-year-old female, BMI 45.1 (HbA1c 5.4%);

Donor #3: 68-year-old female, BMI 26.5 (HbA1c 5.3%);

Donor #4: 38-year-old male, BMI 35.7 (HbA1c 5.6%);

Donor #5: 37-year-old male, BMI 35.6 (HbA1c 5.4%).

### 2.6. Western Blotting

Cells or islets were lysed in RIPA buffer (25 mM Tris, pH 7.5, 100 mM NaCl, 1% Triton X-100, 0.2% deoxycholic acid, 0.1% SDS, and 10 mM EDTA) supplemented with protease and phosphatase inhibitor cocktails. The collected lysates were centrifuged at 12,000× *g* for 15 min at 4 °C, and the supernatants were stored at −80 °C. Protein lysates (5–10 µg) were mixed with gel loading buffer (Invitrogen, Carlsbad, CA, USA) containing 200 mM dithiothreitol, heated at 95 °C for 5 min, and resolved by SDS–PAGE using 4–12% gradient or 12% NuPAGE gels. Proteins were then electrotransferred to nitrocellulose membranes, blocked with 5% bovine serum albumin, and incubated with primary antibodies overnight at 4 °C, followed by incubation with HRP-conjugated secondary antibodies at room temperature for 30 min. Immunoreactive bands were visualized using Bio-Rad Clarity (Carlsbad, CA, USA) or Cytiva ECL (Marlborough, MA, USA).

### 2.7. Mass Spectrometry

Media samples were analyzed by liquid chromatography–mass spectrometry (LC-MS), using an Agilent 1200 liquid chromatograph coupled to a tandem quadrupole mass spectrometer (Agilent 6410, Santa Clara, CA, USA) as previously described [29]. Amino acid concentration was quantified using Agilent MassHunter Analysis software, measuring peak area ratio to the matching calibrated internal standard.

### 2.8. Statistics

**Figure 3:**
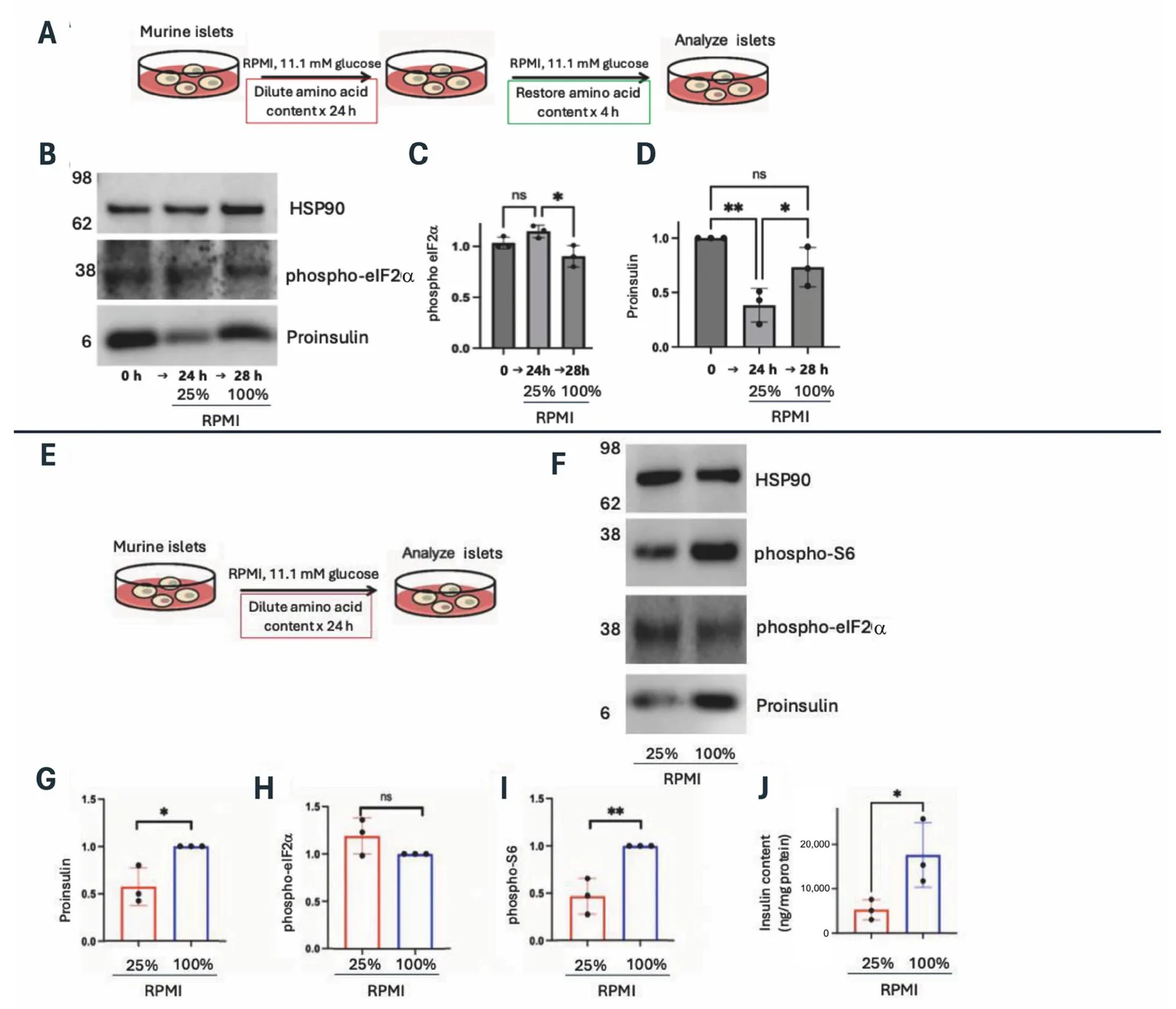
Sensitivity of proinsulin levels to amino acid availability in murine islets. Schema for fasting (**A**,**E**) and re-feeding (**A**) murine islets [pooled islets from two mice in each experiment, n = 3 independent experiments, two using males (squares), one using females (circles)]. Freshly isolated islets were first allowed to recover overnight in complete RPMI-1640 medium containing 11.1 mM glucose (all subsequent steps were also at this glucose concentration). (**B**) Islets were then divided into three sets (35 islets per set). One set of islets was lysed at time 0 h (first lane). Two additional sets of islets (from the same prep) were transferred to micro-tubes with a limiting volume of RPMI medium (2 μL per islet) containing reduced (25%) amino acid content for 24 h (second lane). The third set of islets received an additional 4 h incubation (2 μL per islet) in complete RPMI medium (third lane) before immunoblotting of all samples for phospho-eIF2α and proinsulin (HSP90 is a loading control; molecular weight markers in kDa are indicated). From three independent animal experiments like that shown in (**B**), phospho-eIF2α (**C**) and proinsulin (**D**) normalized to HSP90 were quantified (mean ± sd; one-way repeated-measures ANOVA with the Geisser–Greenhouse correction followed by Tukey’s multiple-comparisons test; * *p* < 0.05; ns = nonsignificant). From these samples, media glucose level dropped only 16% over the initial 24 h incubation, which was restored in the subsequent 4 h incubation. (**F**) Representative immunoblots of proinsulin, phospho-eIF2α, and phospho-S6 following incubation under the conditions shown in panel (**E**). HSP90 is a loading control. From three such independent animal experiments [two mice per experiment, two using females (circles), one using males (squares), 35 islets per condition], quantitation of islet proinsulin (**G**), phospho-eIF2α (**H**), and phospho-S6 (**I**) were each normalized to HSP90 (mean ± sd; two-tailed paired *t*-test; * *p* < 0.05, ** *p* < 0.01; ns = nonsignificant). (**J**) Mature insulin content measured by ELISA in the same islet preps shown in (**G**–**I**) (mean ± SD; two-tailed paired *t*-test; * *p* < 0.05).

**Figure 4:**
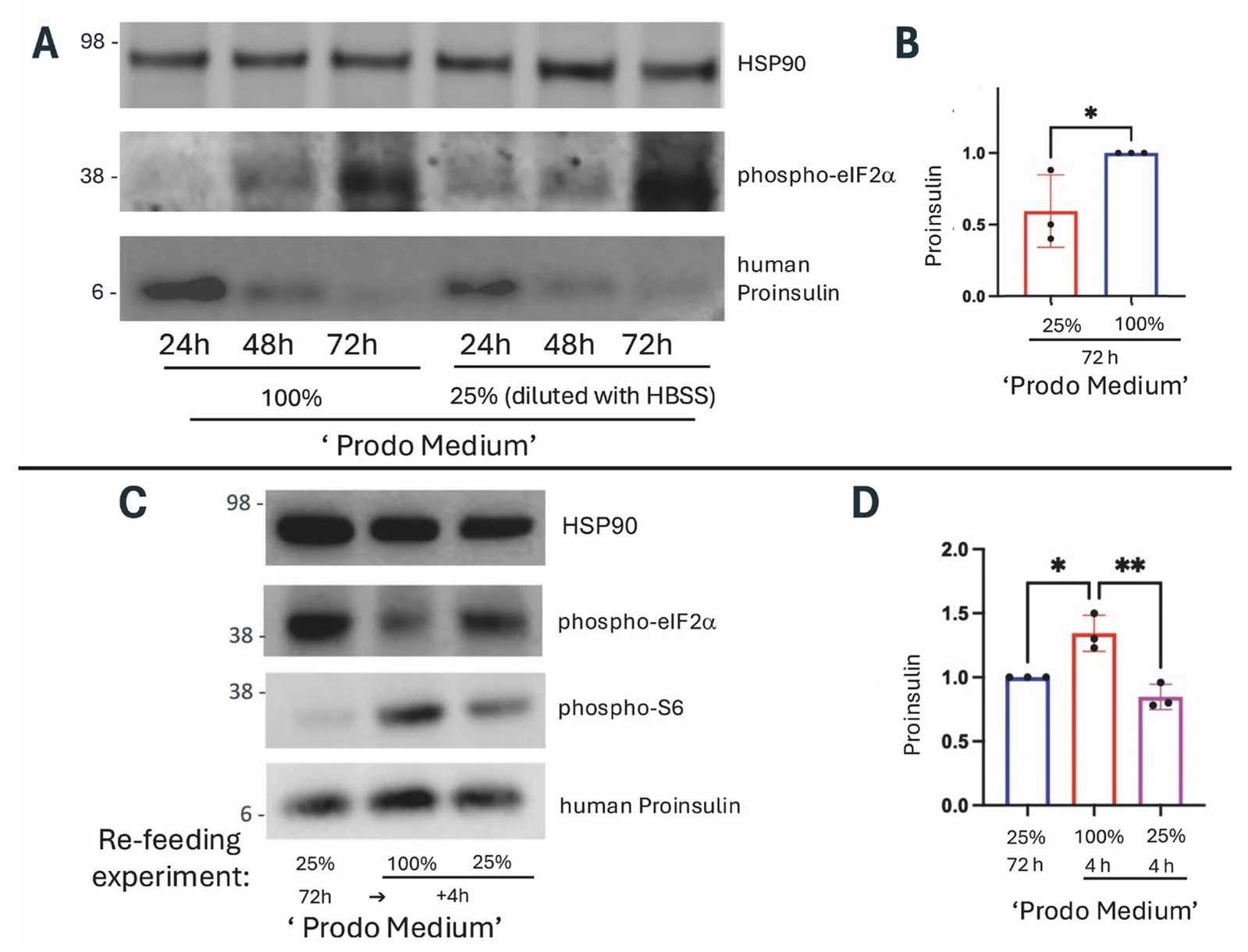
Nutrient availability regulates proinsulin levels in isolated human islets. Isolated human pancreatic islets from three independent nondiabetic donors were obtained from Prodo Labs, maintained in Prodo medium, and then divided among different treatment conditions. (**A**) Representative immunoblots showing human islet phospho-eIF2α and proinsulin levels at different times after the last feeding with the indicated media; HSP90 is a loading control; molecular weight markers in kDa are indicated. To decrease the abundance of amino acids, Prodo medium was diluted with HBSS containing 5.8 mM glucose. (**B**) Quantitation of the relative content of proinsulin levels (normalized to HSP90) in human islets incubated in Prodo medium with diluted or undiluted amino acids (independent donors #1, #2 and #3; two-tailed paired *t*-test; mean ± sd, * *p* < 0.05). (**C**) Representative immunoblotting of human islets after fasting (with diluted amino acids) followed by re-feeding (4 h) with Prodo medium bearing complete or diluted amino acids. The samples were analyzed by reducing SDS-PAGE and immunoblotting for phospho-eIF2α, phospho-S6, and human proinsulin (HSP90 is a loading control; molecular weight markers in kDa are indicated). (**D**) Quantitation of relative proinsulin levels under the conditions shown in panel (**C**) (independent donors #3, #4 and #5; one-way repeated-measures ANOVA with the Geisser–Greenhouse correction followed by Tukey’s multiple-comparisons test; mean ± sd, * *p* < 0.05; ** *p* < 0.01).

Statistical analyses were performed using GraphPad Prism 10 software (San Diego, CA, USA). Comparisons between two groups were analyzed using a two-tailed Student’s *t*-test. For INS1E experiments, comparisons among three or more groups were performed using one-way ANOVA with Tukey’s multiple-comparisons test. Because of a small n-number of experiments, mouse and human islet studies in Figures 3D and 4D were analyzed using one-way repeated-measures ANOVA without assuming sphericity; the Geisser–Greenhouse correction was applied, followed by Tukey’s multiple-comparisons test. Comparisons between two matched conditions were analyzed using two-tailed paired *t*-tests. Data are presented as mean ± SD; a *p*-value < 0.05 was considered statistically significant.

## 3. Results

### 3.1. Fasting and Re-Feeding INS1E Cells

**Figure 1:**
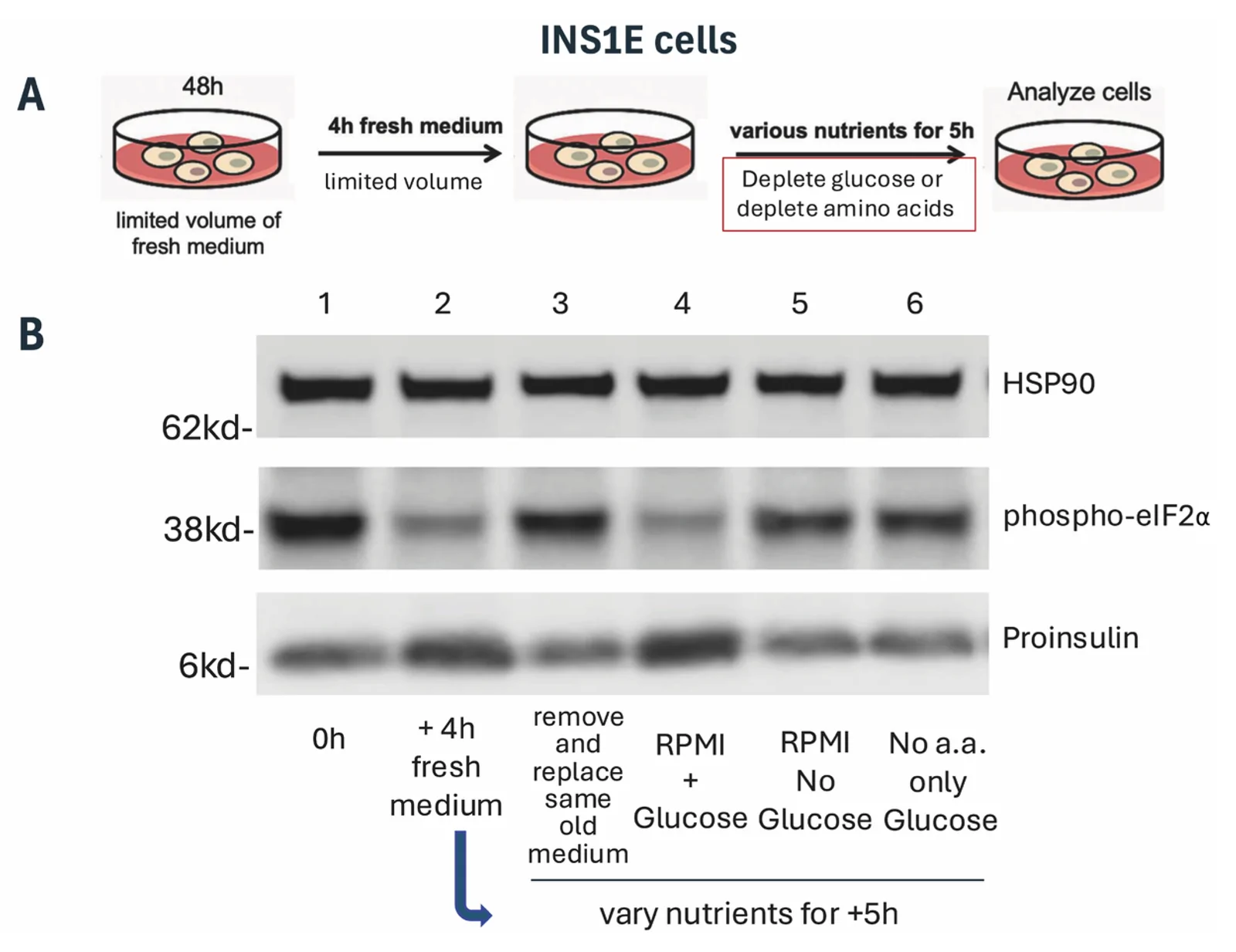
Nutrient availability regulates proinsulin pool size in INS1E cells. (**A**) Experimental schema. In each of three independent replicate cell culture experiments (quantified in Supplemental Figure S2A), INS1E cells cultured in RPMI-1640 medium were fed every 24 h with a limiting volume of medium (90 μL per cm^2^ surface area) until 48 h. (**B**) Twenty-four hours after the last feeding, a subset of cells was lysed at the “zero time” point (lane 1). Another set of cells was then incubated with fresh complete RPMI medium containing 11.1 mM glucose for 4 h (lane 2). In the remaining samples, for an additional 5 h, this medium was then removed and either simply returned to the cells (lane 3) or replaced with media of varying nutrient composition as indicated (lanes 4–6) prior to reducing SDS–PAGE and immunoblot analysis with mouse monoclonal anti-rodent proinsulin and rabbit polyclonal anti-phospho-eIF2α. HSP90 served as a loading control.

To initially examine the effects of fasting and feeding, the INS1E (rat) pancreatic β-cell line was fed 48 h (and again at 24 h) before the start of each experiment with a limited volume of complete RPMI-1640 medium (Figure 1A and Figure S1) (RPMI contains 11.1 mM glucose and amino acid levels that have been previously described [32]). Our protocol is designed to achieve a fasted state of the cells on the day of the experiment (Figure 1B lane 1). INS1E cells were then incubated for 4 h with a limited volume of fresh RPMI, which increased intracellular proinsulin while suppressing phospho-eIF2α (Supplemental Figure S1 at 4 h; Figure 1B lane 2). This was followed by a further re-feeding for five hours with media of various compositions. Simply continuing with the same medium without re-feeding (Supplemental Figure S1) or removing and returning the same spent medium back to the cells (Figure 1B lane 3) resulted in a decline in proinsulin accompanied by re-phosphorylation of eIF2α. Instead, re-feeding complete medium maintained the increase in proinsulin level as well as suppressed phospho-eIF2α (Figure 1B lane 4). In contrast, adding fresh media that either omitted 11.1 mM glucose or omitted the amino acids of RPMI each yielded phospho-eIF2α stress responses, accompanied by an inability to fully restore proinsulin (Figure 1B lanes 5 and 6). These data (quantified in Supplemental Figure S2A) indicate a requirement for both glucose and non-glucose nutrients for optimal proinsulin production.

**Figure 2:**
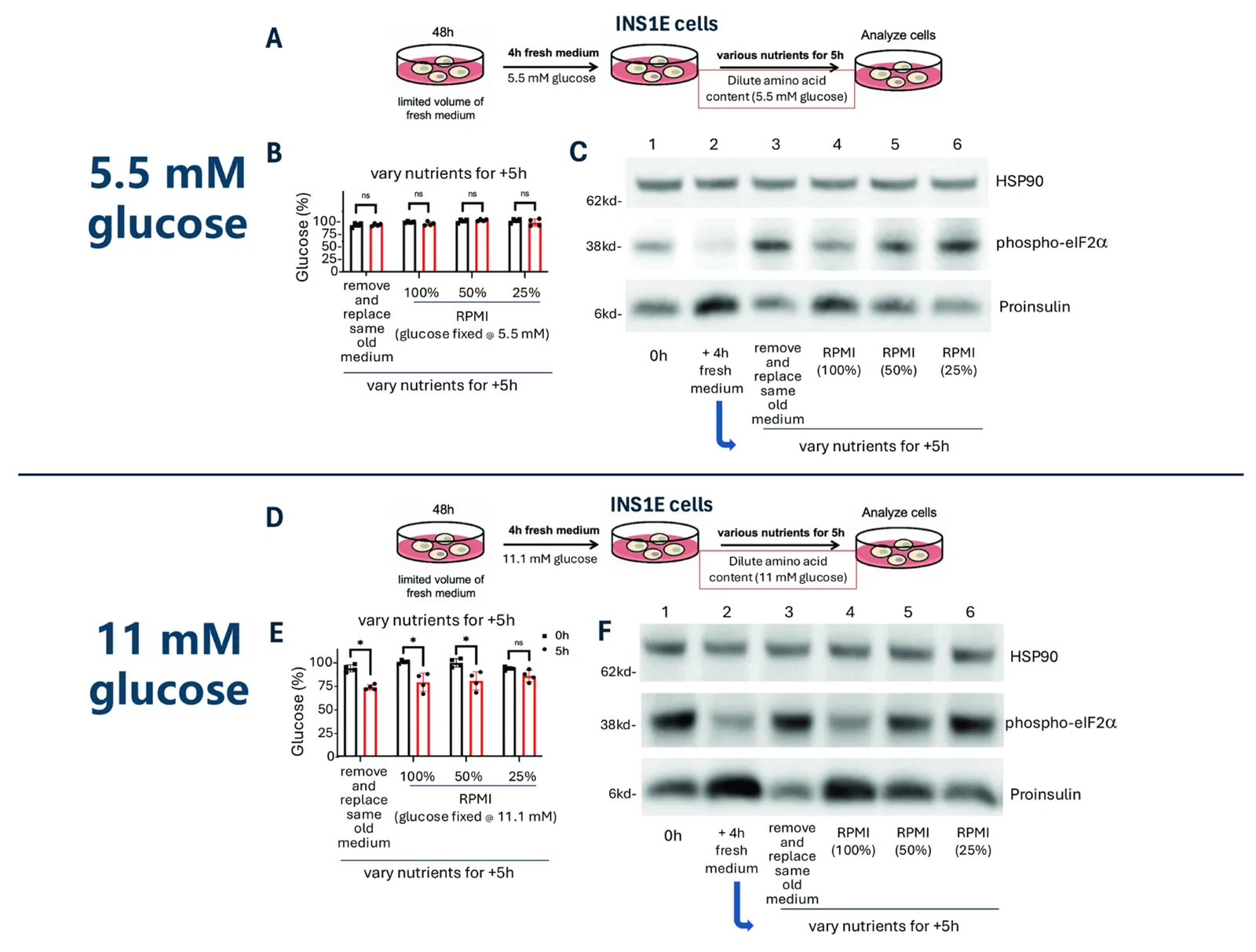
Sensitivity of proinsulin levels to amino acid availability in INS1E cells at two different glucose concentrations. (**A**,**D**) Experimental schema for fasting and re-feeding INS1E (rat) pancreatic β-cells under conditions intended to “clamp” extracellular glucose at either 5.5 mM (**A**) or 11.1 mM (**D**). Cells were fasted by culture with a limiting volume of RPMI, then preincubated for 4 h in fresh medium, and then re-fed for an additional 5 h with media containing graded dilutions of RPMI amino acids (100%, 50%, or 25%) but with glucose undiluted. (**B**,**E**) Extracellular glucose levels before and after the 5 h re-feeding period (n = 4 independent replicate cell culture experiments; mean ± sd, * *p* < 0.05; ns = nonsignificant). (**C**,**F**) Immunoblot analysis of proinsulin and phospho-eIF2α for all of the conditions described above; HSP90 is a loading control; molecular weight markers (kDa) are indicated. Quantitative densitometry is shown in Supplemental Figure S2B,C (n = 3 independent replicate cell culture experiments).

To determine if the sensitivity of proinsulin levels to amino acid availability is linked to extracellular glucose levels, we examined two different glucose concentrations—one approximating euglycemia (or the upper end of the fasting glucose range, 5.5 mM) and another in the post-prandial range (11.1 mM) (Figure 2A,D). At 5.5 mM glucose, re-feeding with medium diluted in buffer to bear only one-half or one-quarter the abundance of RPMI amino acids [31] did not lead to any measurable consumption of extracellular glucose during the five-hour incubation (Figure 2B) but resulted in a progressive decline in proinsulin level with a concomitant increase in phospho-eIF2α (Figure 2C lanes 4–6; quantified in Supplemental Figure S2B). Similarly, at 11.1 mM glucose, the majority of extracellular glucose was not consumed by the β-cells under any incubation conditions (Figure 2E), but proinsulin and phospho-eIF2α levels followed the same pattern as that seen under the lower glucose condition (Figure 2F; quantified in Supplemental Figure S2C). Quantitation of such Western blot data (including phospho-eIF2α levels as well as phospho-S6, phospho-eIF4E, and phospho-AKT levels, discussed below) was not dependent upon the choice of HSP90 as a normalization control for protein loading (Supplemental Figure S3). These findings and prior observations [29] suggest that the sensitivity of β-cell insulin biosynthesis to amino acid availability is similar at both glucose concentrations.

### 3.2. Sensitivity of Proinsulin Level to Amino Acid Availability in Murine and Human Islets

We have shown that proinsulin levels in mouse islets are affected by feeding [29]. To test the impact of amino acid availability, murine islets were incubated for 24 h in either complete RPMI or that diluted to bear a 75% reduction in the abundance of amino acids, with glucose at 11.1 mM (Figure 3A,E). With reduced amino acid availability, there was a diminished proinsulin level and a greater phospho-eIF2α level (Figure 3C,D) accompanied by diminished phosphorylation of ribosomal S6 (Figure 3F–I), an indicator of mTORC1 activity [33]. A sustained decrease in proinsulin content was associated with diminished insulin content (Figure 3J), but the diminished proinsulin content could acutely rebound upon re-feeding complete RPMI medium (Figure 3D).

In isolated human islets from nondiabetic donors, starvation for 24–72 h from the last feeding of Prodo medium caused a dramatic reduction in proinsulin level accompanied by eIF2α phosphorylation (Figure 4A,B). (The composition of Prodo medium is proprietary, but it is amino acid rich and contains 5.8 mM glucose.) A four-hour re-feeding with medium containing only one quarter of the amino acid content (but with extracellular glucose essentially “clamped”, measured and confirmed) could not yield full suppression of phospho-eIF2α nor full rebound of phospho-S6 and proinsulin levels (Figure 4C,D). Together, these data strongly suggest that amino acid availability contributes importantly not only to proinsulin content in β-cell lines but also in both murine and human pancreatic islets.

### 3.3. In β-Cells, Proinsulin Sensitivity to Amino Acid Availability Shows Linkage to mTOR

**Figure 5:**
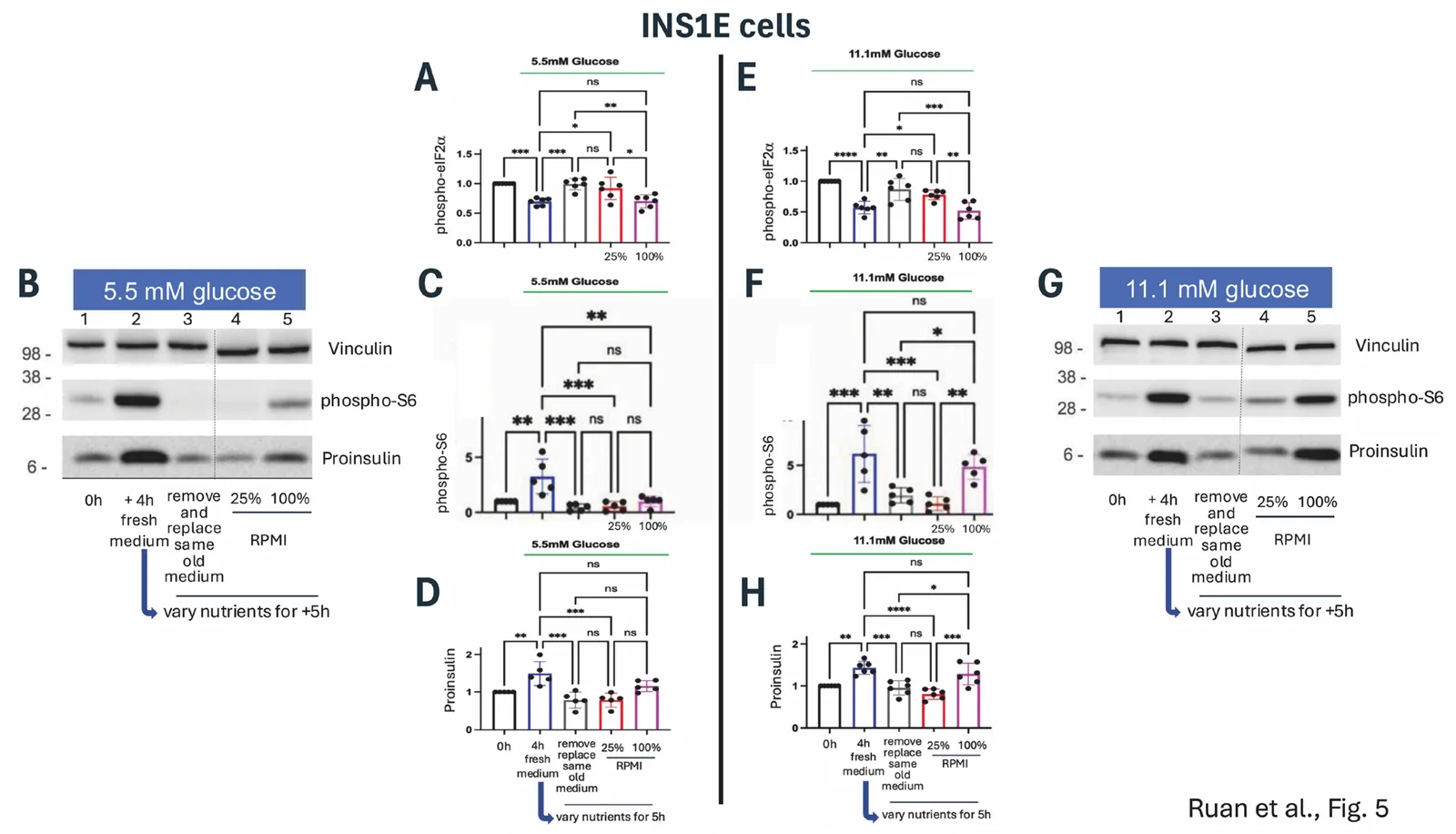
Amino acid availability influences phospho-S6 and proinsulin oppositely from phospho-eIF2α levels in INS1E cells. INS1E cells were subjected to fasting and re-feeding as described in Figure 1, with extracellular glucose maintained at either 5.5 mM (**A**–**D**) or 11.1 mM (**E**–**H**), followed by a 5 h incubation in media of varying amino acid content. (**A**,**E**) Quantification of phospho-eIF2α levels at time 0 (first bar) and, at two different glucose concentrations, after preincubation (4 h) in fresh complete media followed by re-feeding (5 h) under conditions described at the bottom of the figure. Data are expressed relative to the 0 h condition. (**B**,**G**) Representative immunoblots of phospho-S6 and proinsulin from INS1E cells (vinculin is a loading control; molecular weight markers in kDa are indicated) under the described conditions at the two different glucose concentrations. (**C**,**F**) Quantitation of phospho-S6 levels and (**D**,**H**) proinsulin levels (normalized to vinculin) corresponding to the representative immunoblots shown in panels (**B**,**G**) (n = 5 independent replicate cell culture experiments; mean ± SD; * *p* < 0.05, ** *p* < 0.01, *** *p* < 0.001; **** *p* < 0.0001; ns = nonsignificant).

Islets comprise multiple cell types, and as pancreatic α-cells sense amino acids to activate mTORC1 and phosphorylate S6 [34], we returned to INS1E cells in order to test mTOR-mediated responses specific to β-cells. With extracellular glucose essentially “clamped” at either 5.5 mM or 11.1 mM, re-feeding either with spent medium or that containing only one-fourth of the amino acids of complete media resulted in increased phospho-eIF2α and diminished phospho-S6 and diminished proinsulin (Figure 5B,G quantified in Figure 5A and Figure 5E, Figure 5C and Figure 5F, and Figure 5D and Figure 5H, respectively). Re-feeding with the full complement of amino acids resulted in restoration of phospho-S6 and proinsulin levels, particularly under 11.1 mM glucose conditions (Figure 5G lane 5) without any increase in *Ins1*/*Ins2* mRNA. (The re-feeding effect on phospho-S6 and proinsulin observed at 5.5 mM glucose in lane 5 of Figure 5B did not achieve statistical significance in Figure 5C,D) Thus, in response to amino acid availability, these findings in β-cells show a correlation between increased phospho-S6 (a reflection of mTORC1 activation) and increased β-cell proinsulin levels.

**Figure 6:**
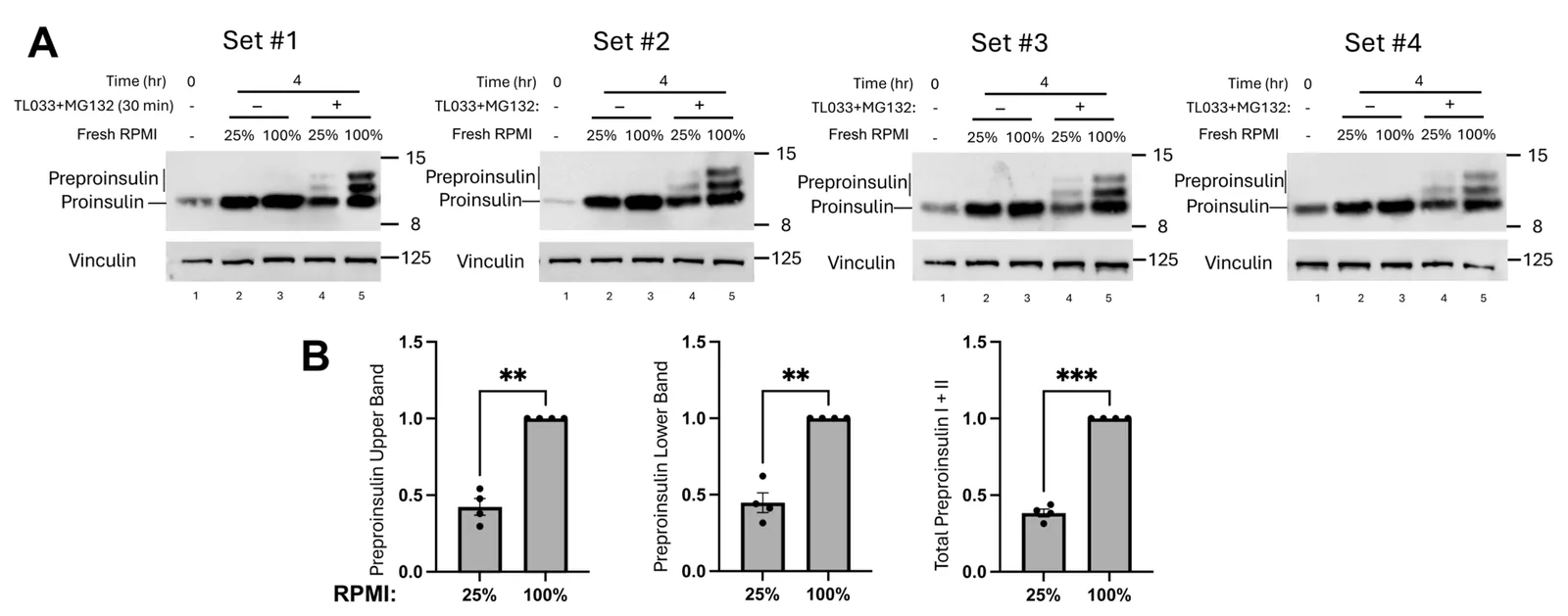
Amino acid availability influences proinsulin biosynthesis in INS1E cells. Cells were cultured in a limited volume of complete RPMI medium (11.1 mM glucose) and fed daily for 48 h and then lysed to establish the baseline (0 h) condition. (**A**) Parallel sets of cells were further incubated for 4 h in fresh RPMI media (with glucose undiluted at 11.1 mM) containing diluted (25%) or complete (100%) amino acids. Where indicated, during the final 30 min prior to cell lysis, cells were treated with the ER translocon inhibitor (TLO33, 10 μM) plus proteasome inhibitor (MG132, 10 μM) to trap newly synthesized preproinsulin-1 and preproinsulin-2 (two distinct upper bands). Upper panels: immunoblots with anti-proinsulin antibody, which also detects preproinsulin; lower panels: vinculin is a loading control; molecular weight markers (in kDa) are indicated at right. (**B**) Quantitation of preproinsulin bands in the concurrent presence of translocon + proteasome inhibitors, normalized to loading control. N = 4 independent replicate cell culture experiments (mean ± sd, ** *p* < 0.01; *** *p* < 0.001).

Using a protocol similar to Figure 2F lanes 1 and 2, we directly examined the impact on proinsulin biosynthesis of RPMI bearing a 75% reduction in the abundance of amino acids (with glucose held at 11.1 mM). For this, we used a recently developed method that involves a brief treatment with an ER translocon inhibitor combined with a proteasome inhibitor in order to trap preproinsulin on the cytosolic side of the ER while simultaneously preventing its proteasomal degradation [35]. In the last 30 min of a 4 h feeding with complete 100% RPMI, preproinsulin-1 and -2 were detected only upon the concurrent addition of translocon and proteasome inhibitors (Figure 6A, last lane of each repeat experiment). In media bearing a 75% reduction in the abundance of amino acids, proinsulin biosynthesis (detected as preproinsulin) was significantly decreased (quantified in Figure 6B), whereas global protein synthesis appeared less affected (Supplemental Figure S4).

**Figure 7:**
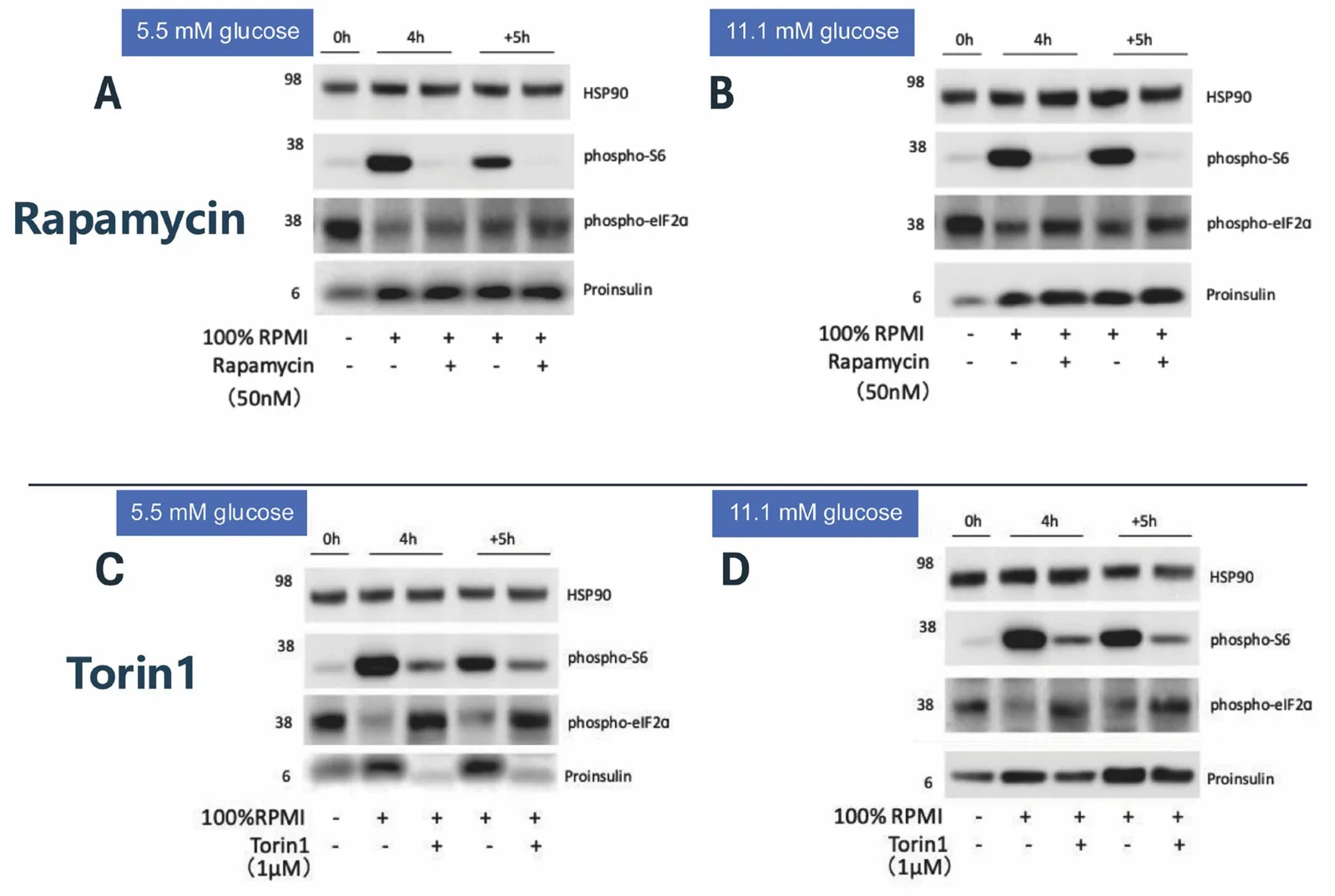
Effects of distinct mTOR inhibitors on phospho-S6 and proinsulin levels during fasting and re-feeding in INS1E cells. (**A**,**B**) In each of three replicate experiments, INS1E cells cultured in complete RPMI medium were lysed at baseline (0 h) or after preincubation with complete RPMI (100% amino acids, 4 h) ± rapamycin (50 nM) or were treated for the first time ± rapamycin during an additional 5 h incubation either at 5.5 mM (**A**) or 11.1 mM glucose (**B**). Representative immunoblots of phospho-S6, phospho-eIF2α, and proinsulin are shown (HSP90 is a loading control; molecular weight markers in kDa are indicated). (**C**,**D**) Cells were treated and analyzed identically to those in panels (**A**,**B**) except that Torin1 (1 μM) was used instead of rapamycin.

To test the role of mTOR, we first treated INS1E cells (fed in the presence of all the amino acids of RPMI) with rapamycin either during the 4 h re-feeding incubation and/or during additional hours of re-feeding. When extracellular glucose was essentially “clamped” at 11.1 mM, the presence of rapamycin fully suppressed phospho-S6 via mTORC1 but did not prevent the increase in proinsulin triggered by feeding complete medium (Figure 7)—indeed, this result was observed across a range of rapamycin concentrations (Supplemental Figure S5A). The suppression of phospho-S6 was similar when extracellular glucose was essentially “clamped” at 5.5 mM (Figure 7A and Figure S5B upper left graph).

We have proposed that the amino acid-stimulated increase in proinsulin level is driven by upregulated proinsulin synthesis (Figure 6) [29], which has been posited to be mediated mostly by cap-dependent (eIF4F complex-regulated) translation under physiological conditions [36,37,38,39]. In many cell types, rapamycin inhibits mTORC1-mediated ribosomal S6 phosphorylation while not effectively inhibiting cap-dependent translation [40]; and rapamycin has been found to be unable to inhibit proinsulin synthesis in β-cells [41] even though mTORC1 is thought to increase β-cell/islet insulin synthesis [42]. The increase in INS1E proinsulin content in the presence of 100% amino acid content was not accompanied by any increase in AKT-phosphoSer473 (Supplemental Figure S3) that has been attributed to the activity of mTORC2 [43]. By contrast, Torin1, up to a dose of 1 µM, selectively inhibits the catalytic activity of the mTOR kinase [44], blocking most mTORC1 and mTORC2 activity [45,46,47], including dose-dependent inhibition of the phosphorylation of eIF4E-BP1 (Supplemental Figure S6, compare panels A and B). Treatment with Torin1 did not allow phospho-eIF2α levels to precipitously drop upon re-feeding (Figure 7C,D; Supplemental Figure S5B lower panels)—potentially attributable to de-repressing the GCN2 kinase by PP6C activity and/or by blocking the entry (efflux) of amino acids to the cytosol from lysosomes upon mTORC1 inhibition [48,49]. Altogether, the data in Figure 7C,D suggest that when essentially “clamped” at either extracellular glucose concentration, rapamycin-resistant, Torin1-sensitive mTOR signaling contributes to amino acid-dependent stimulation of β-cell proinsulin levels upon re-feeding.

In most of our studies, we diluted RPMI fourfold to decrease amino acid abundance, and in so doing we also diluted vitamins and other components. We note that none of the non-glucose components of RPMI other than amino acids have any known acute or sub-acute effects on proinsulin biosynthesis, but to confirm this, we tested our standard protocol using amino acid-free RPMI that contains all other components (Supplemental Figure S7A). In this case too, the aforementioned regulation of proinsulin (Supplemental Figure S7B), phospho-eIF2α (Supplemental Figure S7C,D), phospho-4E-BP1 (Supplemental Figure S7E) and phospho-S6 (Supplemental Figure S7F) was observed.

It has been established that Min6 (mouse) β-cells avidly and selectively consume more of certain amino acids (such as glutamine and serine) from the extracellular medium (DMEM) than others [29,50]. However, what has not been reported before is that from the same cells under the same conditions, mass spectrometry analysis of the medium revealed that six nonessential amino acids were actually exported from cells to the medium—particularly notable are proline and alanine plus glutamic and aspartic acid (Supplemental Figure S8A). We also examined INS1E β-cells re-fed with a limited supply of amino acids (one quarter of that normally contained in RPMI) for 5 h. Despite differences in culture medium, cell line and species of origin, as well as differences in experimental protocol [29], the same six nonessential amino acids were exported to the medium, most notably the release of proline and alanine plus glutamic and aspartic acid (Supplemental Figure S8B)—the latter of which are known to be generated in islets with carbons that can be traced from glucose metabolism (i.e., cataplerosis [51]). Such behavior suggests the possibility that β-cells might engage amino acid exchangers such as LAT1 and other bi-directional plasma membrane transporters (whose activity can be coupled to mTOR activation [52,53,54]) in order to incorporate desired amino acids from the extracellular space.

**Figure 8:**
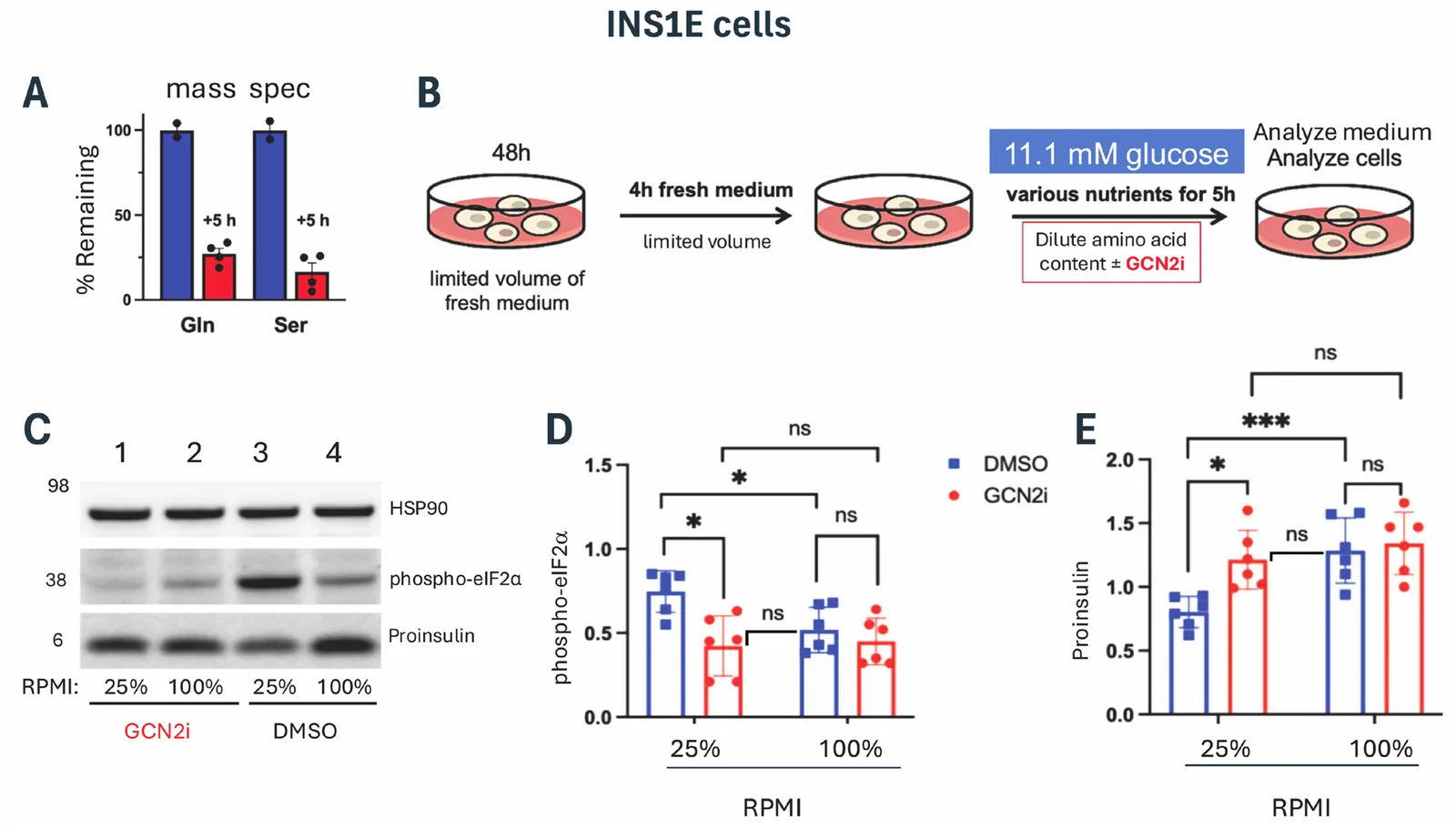
Amino acid consumption and sensing in INS1E cells involved GCN2 kinase activity. (**A**) INS1E cells were fed with RPMI bearing amino acids diluted to 25% (11.1 mM glucose) for 5 h. Mass spectrometry analysis measured the consumption of extracellular glutamine (Gln) and serine (Ser) from the initially fed levels in the 25% RPMI medium. (**B**) Experimental design to test amino acid sensing by GCN2. (**C**) INS1E cells were subjected to fasting and were re-fed for 5 h with either 25% amino acid dilution or complete RPMI (glucose fixed at 11.1 mM) ± GCN2 inhibitor (GCN2i 10 μM; DMSO was a vehicle). Representative immunoblots of phospho-eIF2α and proinsulin (HSP90 was a loading control; molecular weight markers in kDa are indicated). (**D**,**E**) From 6 independent replicate cell culture experiments, quantitation of relative phospho-eIF2α (**D**) and proinsulin (**E**), each normalized to HSP90 (mean ± SD. * *p* < 0.05, *** *p* < 0.001; ns = nonsignificant).

As observed in Min6 cells [29], INS1E β-cells consumed the majority of extracellular glutamine and serine upon re-feeding with a limited supply of amino acids (Figure 8A). Intracellular deficiency of these or other amino acids can trigger the activity of GCN2 to increase phospho-eIF2α, as was observed in Figure 1, Figure 2, Figure 3, Figure 4 and Figure 5. In INS1E β-cells re-fed with a limited supply of amino acids, the increase in phospho-eIF2α (Figure 8C lane 3) was blocked by the addition of a GCN2 kinase inhibitor (Figure 8C lane 1, quantified in the first two bars of Figure 8D), and this was accompanied by increased proinsulin (first two bars of Figure 8E) even in the setting of ongoing amino acid deficiency. As re-feeding with full-strength RPMI medium also inhibited the increase of phospho-eIF2α (Figure 1B lane 4, Figure 2C,F lane 4, Figure 8C lane 4, and quantified in Figure 5A,E), these data confirm that suppression of GCN2 activity also contributes to amino acid-dependent translational stimulation of β-cell proinsulin levels [29].

**Figure 9:**
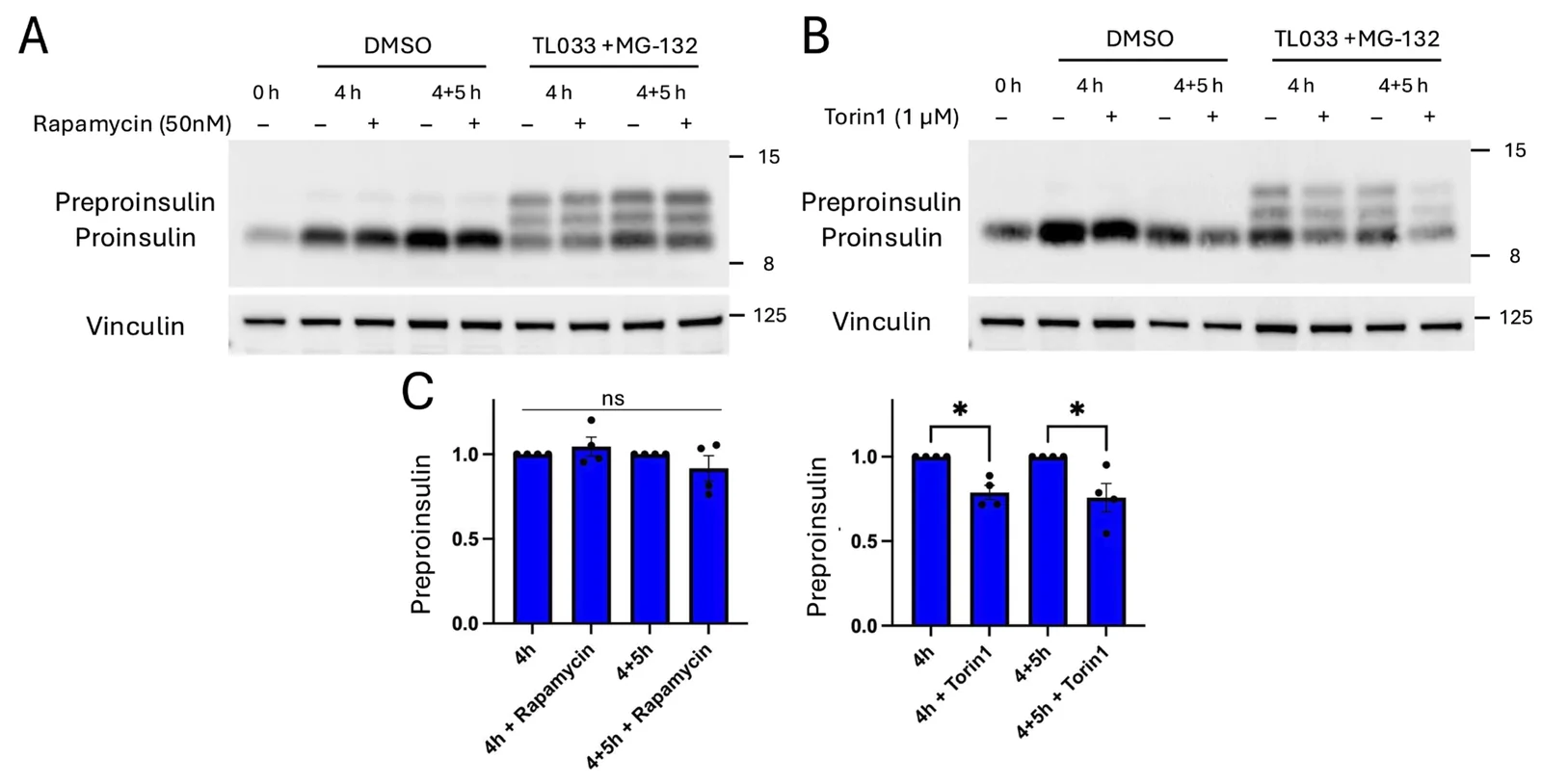
mTOR activity is required for proinsulin biosynthesis in INS1E cells. (**A**,**B**) In each of four independent replicate cell culture experiments, cells were cultured in a limited volume of complete RPMI medium (11.1 mM glucose) and fed daily for 48 h and lysed to establish the baseline (0 h) condition. Additional cells were then preincubated with a limited volume of fresh complete RPMI for 4 h ± mTOR inhibitors or were re-fed with fresh media ± mTOR inhibitors for a further 2 h period as in Figure 7. (**A**) Rapamycin treatment. (**B**) Torin1 treatment. Where indicated, cells were treated with the ER translocon inhibitor TL033 (10 μM) concurrently with the proteasome inhibitor MG132 (10 μM) for the final 30 min prior to cell lysis to trap newly synthesized preproinsulin. Upper blots: anti-proinsulin antibody; lower blots: vinculin is a loading control; molecular weight markers (in kDa) are indicated at the far right. (**C**) Quantitative densitometry of the upper preproinsulin band (normalized to loading control) from the four independent cell culture experiments (mean ± SD. * *p* < 0.05; ns = nonsignificant).

In support of the idea that amino acid-supported, rapamycin-resistant mTOR activity provides translational stimulation of β-cell proinsulin levels, we found that in RPMI medium containing the full complement of amino acids, treatment with rapamycin did not block preproinsulin biosynthesis (Figure 9A), but Torin1 treatment inhibited the appearance of preproinsulin (Figure 9B). These data suggest that amino acids delivered to β-cells via the extracellular environment (bloodstream, interstitial fluid, etc.) stimulate proinsulin synthesis both by mTOR activation and GCN2 suppression [55,56,57,58].

## 4. Discussion

Many tissues respond to circadian-based feeding/fasting cycles. For pancreatic β-cells, post-prandial cycles involve not only a rise in the levels of blood glucose but also that of multiple nutrients. In fact, CGM studies in normal humans under eating conditions of everyday life show that minimum–maximum blood glucose variation across the 24 h day is only ~2-fold (~75–150 mg/dL) [59], with surprisingly little effect of exercise [60]. In contrast, blood levels of various amino acids fluctuate with every feeding and fasting cycle and may vary more widely [61]. Amino acids (and other nutrients/metabolites) from the circulation are taken up and metabolically sensed through nutrient-regulated signaling pathways in all tissues (including β-cells). However, thus far our understanding of the role of these amino acids in maintaining the proinsulin content that leads to insulin biosynthesis is poorly developed.

It has been highlighted that in countries from Asia and Africa, the fraction of underweight or normal-weight individuals with diabetes ranged from 24 to 66% [28]. These authors also reviewed that underweight individuals with diabetes tend to exhibit a significant decrease in HOMA-β (a fasting measure of circulating insulin) as well as insulinogenic index (a stimulated measure of circulating insulin). In a study of new-onset “T2D” patients in Uganda, 22% of such patients were underweight and autoantibody-negative, with measured insulin deficiency [24] as judged by multiple measures of pancreatic secretory function [25]. Similar phenotypes were observed in lean “T2D” patients in rural India [26]. As reviewed by Wadivkar et al., such individuals—now described as having type 5 diabetes (T5D)—are notable in that they consume less protein than individuals with T1D or T2D, and the authors cited a World Health Organization-defined subgroup of Protein-Deficient Pancreatic Diabetes [62]. Prajitno and Sutanto have argued that T5D is caused by severe (non-autoimmune) insulin deficiency secondary to protein–calorie malnutrition, which directly compromises pancreatic β-cell function by reducing the availability of amino acids required for insulin biosynthesis [23]. Although controversial [63], a consensus statement reports that the pathophysiology of T5D is one involving a substantial impairment of pancreatic insulin with normal hepatic and peripheral insulin sensitivity, an absence of ketoacidosis, and no islet cell autoantibodies [27]. A recent review urges further research to understand the role of undernutrition [22].

Eizirik and colleagues noted that in rodents, lower protein diets promote greater susceptibility to diabetes [64], and over the course of a week, mature insulin from murine islets (total of cells + media combined) dropped from 122 ng/islet with supplementation of a cocktail of all amino acids to 60.9 ng/islet without amino acid supplementation [65]. Similarly, another group studied rats 3 weeks of age that were fed a low-protein diet (compared to control) for three further weeks: electron microscopy of islet β-cells [66] suggests the presence of insulin microgranules, which we and others have found to be associated with markedly decreased β-cell insulin content [67,68,69].

Here we have found that upon amino acid limitation, isolated murine islets exhibit a state of suppressed mTOR activity with active phosphorylation of eIF2α. Similar results were observed in isolated human islets, although thus far we have tested only a relatively small number of donors with an average BMI of 35 and have used a proprietary medium (i.e., unpublished amino acid concentrations). Nevertheless, for both murine and human islets, subsequent re-feeding can reverse the observed signaling phenotypes, provided that full concentrations of amino acids are restored, which allows islet proinsulin to rebound to levels that support maintenance of insulin content. Of course, islets comprise several different cell types. Nevertheless, we believe that the signaling pathways regulating mTOR and phospho-eIF2α are both critically active in β-cells, as they are phenocopied in INS1E cells. Interestingly, these results are similar but more robust at 11.1 mM glucose than at 5.5 mM glucose, consistent with a crosstalk in amino acid-dependent and glucose-dependent signaling for proinsulin biosynthesis.

We have found that two different pancreatic β-cell lines consume a large fraction of extracellular glutamine and serine while consuming only a small fraction of extracellular glucose. At the same time, these cells are actually exporting several nonessential amino acids into the extracellular space (Supplemental Figure S6) [29]. In β-cells, deficiency of intracellular amino acids leads to signaling via GCN2, driving eIF2α phosphorylation, which suppresses proinsulin levels. In contrast, the fate of exported amino acids is unknown, and there is at least a hypothetical possibility of swapping some amino acids between β-cells and other islet cells such as α-cells, which may be an important consideration for further study. We hypothesize that in the setting of amino acid limitation in β-cells, increased phosphorylation of eIF2α could function as one of two key signaling pathways lowering insulin content in patients or animal models with T5D—although more work will need to be performed in vivo. The phosphorylation of eIF2α is part of the integrated stress response that includes upregulation of amino acid transporters [50], and in many stressed cells, glutamine in particular is a critical anaplerotic fuel source [70], while serine is an important regulator of mitochondrial metabolism [71].

An additional potential explanation for how a lack of available amino acids may lead to β-cell insulin deficiency in T5D is via diminished mTOR activity resulting in impaired eIF4F complex assembly that in turn limits initiation of cap-dependent translation [72]. Upon β-cell re-feeding, addition of rapamycin to inhibit mTORC1 could fully suppress phospho-S6, yet this does not appear to be an important factor in recovery of proinsulin levels [41]. Unlike rapamycin, Torin1 treatment does result in dose-dependent inhibition of eIF4E-BP1 phosphorylation (Supplemental Figure S6) and inhibits proinsulin biosynthesis stimulated by amino acids. As amino acid insufficiency is known to result in limiting the phosphorylation of eIF4E-BP1 (this report and [72]), which affects cap-dependent (eIF4F complex-regulated) translation [36,37,38,39], proinsulin levels may be affected by this mechanism.

In most of our studies, we diluted RPMI, which also dilutes vitamins and other components. However, similar results are obtained when only amino acids are limited (Supplemental Figure S7), and similar results in β-cells are also obtained when amino acids are ultimately consumed from undiluted media (Supplemental Figure S1) [29].

A limitation of the present study is that it did not include in vivo protein-restriction data from whole animals or human subjects. Future studies along these lines are important, as our in vitro starvation model might not reflect the signaling alterations that occur in the β-cells of humans upon physiological fasting conditions or in patients with undernutrition-associated diabetes. β-cells or islets incubated in culture medium in which nutrients are limiting are only model systems that cannot represent the full complexity of the in vivo pancreatic environment.

Nevertheless, it seems plausible that the behaviors observed in this study could contribute to the lowering of insulin content in patients (and animal models) with T5D.

## 5. Conclusions

Although further confirmation will be needed from in vivo studies, altogether, our data support that optimal insulin biosynthesis in pancreatic β-cells requires food-triggered amino acid-dependent signals. Such findings raise the question of whether deficiency of amino acid-stimulated proinsulin biosynthesis could be a driving factor in T5D, which can be treated with interventions that increase protein intake [73,74].

## Data Availability

The original contributions presented in this study are included in the article/Supplementary Materials. Further inquiries can be directed to the corresponding author.

## Supplementary Materials

The following supporting information can be downloaded at: https://www.mdpi.com/article/10.3390/cells15171584/s1, Figure S1. Time-dependent regulation of proinsulin levels upon fasting and re-feeding in INS1E β-cells. Prior to the start of the experiment, INS1E cells cultured in RPMI-1640 medium were fed every 24 h with a limiting volume of medium (90 μL per cm^2^ surface area) until 48 h. One set of cells was lysed at the “zero time” point (in triplicate). The remaining cells were re-fed with fresh complete RPMI medium (11.1 mM glucose) in a limiting volume and lysed at the indicated times (4, 8, 12 h). The cells were lysed and resolved by reducing SDS-PAGE and analyzed by immunoblotting with antibodies against proinsulin and phospho-eIF2α (HSP90 is a loading control; molecular weight markers in kDa are indicated). Figure S2. Quantitative densitometry analysis of experiments shown in Figure 1 and Figure 2C,F. (A) Phospho-eIF2a and proinsulin levels (normalized to loading control) from Figure 1 (n = 3 independent replicate cell culture experiments; mean ± sd, * *p* < 0.05; ** *p* < 0.01; ns = non-significant). (B,C) Phospho-eIF2a and proinsulin levels (normalized to loading control) from Figure 2C and Figure 2F, respectively (n = 3 independent replicate cell culture experiments; mean ± sd, * *p* < 0.05; ** *p* < 0.01; ns = non-significant). Figure S3. Signaling pathway activation upon fasting and re-feeding in INS1E β-cells. Prior to the start of the experiment, INS1E cells cultured in RPMI-1640 medium were fed every 24 h with a limiting volume of complete RPMI medium containing 11.1 mM glucose (90 μL per cm^2^ surface area), until 48 h. Four independent replicate sets of INS1E cells were cultured; in each experiment, cells were lysed at baseline (0 h) and after a 4 h incubation in fresh medium containing either 25% or 100% of RPMI amino-acids. (A) Lysates were resolved by SDS-PAGE and immunoblotting for the listed antigens; HSP90 and Vinculin are both loading controls; eIF2a is a control for phospho-eIF2a; AKT is a control for phospho-AKT-Ser473; molecular weight markers in kDa are indicated at *right*. (B–F) Quantitative densitometry from the samples shown in (A), normalized to various controls; mean ± sd, * *p* < 0.05; ** *p* < 0.01; *** *p* < 0.001; ***** *p* < 0.0001; *p* ≥ 0.05 is non-significant). Figure S4. Effect of limited amino-acid availability on general protein synthesis in INS1E cells. Prior to the start of the experiment, INS1E cells cultured in RPMI-1640 medium were fed every 24 h with a limiting volume of complete RPMI medium containing 11.1 mM glucose (90 μL per cm^2^ surface area), until 48 h. Cells were then cultured in reduced amino acid (25%), full amino acid (100%), or full amino acid plus 500 nM Torin1 for 4 h. During the last 10 min of the 4 h incubation, puromycin (10 µg/mL) was added to lanes 2–4. The cells were lysed, resolved by SDS-PAGE and immunoblotting with anti-puromycin mAb PMY-2A4 (DSHB, Iowa City, IA, USA). Vinculin was used as a loading control. The bottom right panel shows newly-synthesized puromycinylated proteins from full-lane length densitometry (normalized to vinculin), quantified from n = 3 independent, replicate cell culture experiments. Data are presented as mean ± SD; statistics were performed using one-way ANOVA with Tukey’s multiple-comparisons test and no statistically significant differences were observed (25% RPMI *v* Torin1 *p* = 0.72; 25% RPMI *v* 100% RPMI *p* = 0.18; 100% RPMI *v* Torin1 *p* = 0.46). Figure S5. Rapamycin suppresses phospho-S6 although it does not impair the proinsulin response to feeding in INS1E cells. (A) Prior to the start of the experiment, INS1E cells cultured in RPMI-1640 medium were fed every 24 h with a limiting volume of complete RPMI medium containing 11.1 mM glucose (90 μL per cm^2^ surface area), until 48 h. Cells were lysed at baseline (0 h) and after preincubation in fresh complete RPMI medium for 4 h or following an additional 5 h feeding period. Rapamycin was added either during the preincubation period or during the subsequent 5 h re-feeding period, at the indicated concentrations. The cells were lysed and resolved by reducing SDS-PAGE and analyzed by immunoblotting with antibodies against phospho-eIF2α, phospho-S6, and proinsulin (HSP90 is a loading control; molecular weight markers in kDa are indicated). (B) Quantitative densitometry of phospho-S6 (normalized to loading control) and phospho-eIF2a (normalized to eIF2a) from n = 3 independent replicate cell culture experiments like those shown in Figure 7A–D. Figure S6. Dose-response of mTOR inhibitors on proinsulin levels. (A) Prior to the start of the experiment, INS1E cells cultured in RPMI-1640 medium were fed every 24 h with a limiting volume of complete RPMI medium containing 11.1 mM glucose (90 μL per cm^2^ surface area), until 48 h. INS1E were lysed at baseline (0 h) or after 4 h incubation in fresh complete RPMI medium (100% amino-acids) in the presence of the indicated concentrations of Torin1. (B) Cells were treated identically to those in panel (A), except that rapamycin was used at the indicated concentrations. The cells were lysed and resolved by reducing SDS-PAGE and analyzed by immunoblotting with antibodies against phospho-S6, phospho-4E-BP1 and proinsulin (HSP90 is a loading control; molecular weight markers in kDa are indicated at *left*). Figure S7. Proinsulin levels are regulated by amino-acid availability in pancreatic β-cells. The experimental schema was similar to that shown in Figure 2D. Cells were fasted by culture with a limiting volume of RPMI, then “preincubated” for 4 h in a limiting volume of fresh medium, and then re-fed for an additional 5 h with media containing graded dilutions of RPMI amino-acids (0%, 25%, or 100%)—all at 11.1 mM glucose and with all other components of RPMI held constant. (A) Immunoblot analysis of the indicated proteins under all of the conditions described above; Vinculin is the loading control; molecular weight markers (kDa) are indicated. (B–F) Quantitative densitometry from n = 4 independent replicate cell culture experiments (in panel (C) phospho-eIF2a is normalized to the loading control and in panel (D) phospho-eIF2a is normalized total eIF2a). Figure S8. β-cells export selected amino-acids from the cells to the extracellular environment. (A) Min6 (mouse-derived) β-cells were fed complete DMEM medium (25 mM glucose) and media collected after 1 or 8 h as described in [29]. Amino-acid concentrations in the collected media were quantified by LC–MS as described in [29]. From five independent wells, replicates were collected at both time points, and the concentration for each amino-acid is shown in the first set of bars (1 h) and the second set of bars (8 h), demonstrating an increase of amino-acid concentration in the media. (B) INS1E (rat-derived) β-cells were subjected to the fasting/re-feeding protocol shown in the schema above and incubated in diluted RPMI medium (25% amino-acid content at 11.1 mM glucose) for 5 h. The fold-increase of concentration of each amino-acid in the media from the start (duplicates, right out of the stock bottle) and end of the 5 h incubation (n = 4 independent replicate wells).
