# Supplementary material for: Beta Cell Proinsulin Response to Amino Acid Availability
Source: Cells. 2026 Aug 31;15(17):1584. doi: 10.3390/cells15171584 (PMC13565673; doi:10.3390/cells15171584)
Supplement: Supplementary file 1 [file cells-15-01584-s001.zip › cells-4153652-for XML-supplementary.pdf]

Supplemental Figure Legends

Figure S1. Time-dependent regulation of proinsulin levels upon fasting and re-feeding in INS1E β-cells. Prior to the start of the experiment, INS1E cells cultured in RPMI-1640 medium were fed every 24 h with a limiting volume of medium (90 μL per cm² surface area) until 48 h. One set of cells was lysed at the "zero time" point (in triplicate). The remaining cells were re-fed with fresh complete RPMI medium (11.1 mM glucose) in a limiting volume and lysed at the indicated times (4, 8, 12 h). The cells were lysed and resolved by reducing SDS-PAGE and analyzed by immunoblotting with antibodies against proinsulin and phospho-eIF2α (HSP90 is a loading control; molecular weight markers in kDa are indicated).

Figure S2. Quantitative densitometry analysis of experiments shown in Figures 1, 2C, and 2F. **(A)** Phospho-eIF2α and proinsulin levels (normalized to loading control) from Figure 1 (n = 3 independent replicate cell culture experiments; mean ± sd, **p* < 0.05; ***p* < 0.01; ns = non-significant). **(B, C)** Phospho-eIF2α and proinsulin levels (normalized to loading control) from Figure 2C and 2F, respectively (n = 3 independent replicate cell culture experiments; mean ± sd, **p* < 0.05; ***p* < 0.01; ns = non-significant).

Figure S3. Signaling pathway activation upon fasting and re-feeding in INS1E β-cells. Prior to the start of the experiment, INS1E cells cultured in RPMI-1640 medium were fed every 24 h with a limiting volume of complete RPMI medium containing 11.1 mM glucose (90 μL per cm² surface area), until 48 h. Four independent replicate sets of INS1E cells were cultured; in each experiment, cells were lysed at baseline (0 h) and after a 4h incubation in fresh medium containing either 25% or 100% of RPMI amino-acids. **(A)** Lysates were resolved by SDS-PAGE and immunoblotting for the listed antigens; HSP90 and Vinculin are both loading controls; eIF2α is a control for phospho-eIF2α; AKT is a control for phospho-AKT-Ser473; molecular weight markers in kDa are indicated at *right*. (**B-F**) Quantitative densitometry from the samples shown in A, normalized to various controls; mean ± sd, **p* < 0.05; ***p* < 0.01; ****p* < 0.001; ******p* < 0.0001; *p* ≥ 0.05 is non-significant).

Figure S4. Effect of limited amino-acid availability on general protein synthesis in INS1E cells. Prior to the start of the experiment, INS1E cells cultured in RPMI-1640 medium were fed every 24 h with a limiting volume of complete RPMI medium containing 11.1 mM glucose (90 μL per cm² surface area), until 48 h. Cells were then cultured in reduced amino acid (25%), full amino acid (100%), or full amino acid plus 500 nM Torin1 for 4h. During the last 10 min of the 4 h incubation, puromycin (10 μg/mL) was added to lanes 2-4. The cells were lysed, resolved by SDS-PAGE and immunoblotting

with anti-puromycin mAb PMY-2A4 (DSHB, Iowa City IA, USA). Vinculin was used as a loading control. The bottom right panel shows newly-synthesized puromycinylated proteins from full-lane length densitometry (normalized to vinculin), quantified from n = 3 independent, replicate cell culture experiments. Data are presented as mean ± SD; statistics were performed using one-way ANOVA with Tukey's multiple-comparisons test and no statistically significant differences were observed (25% RPMI *v* Torin1 *p*=0.72; 25% RPMI *v* 100% RPMI *p*=0.18; 100% RPMI *v* Torin1 *p*=0.46).

Figure S5. Rapamycin suppresses phospho-S6 although it does not impair the proinsulin response to feeding in INS1E cells. (A) Prior to the start of the experiment, INS1E cells cultured in RPMI-1640 medium were fed every 24 h with a limiting volume of complete RPMI medium containing 11.1 mM glucose (90 µL per cm² surface area), until 48 h. Cells were lysed at baseline (0 h) and after preincubation in fresh complete RPMI medium for 4 h or following an additional 5 h feeding period. Rapamycin was added either during the preincubation period or during the subsequent 5 h re-feeding period, at the indicated concentrations. The cells were lysed and resolved by reducing SDS-PAGE and analyzed by immunoblotting with antibodies against phospho-eIF2α, phospho-S6, and proinsulin (HSP90 is a loading control; molecular weight markers in kDa are indicated). **(B)** Quantitative densitometry of phospho-S6 (normalized to loading control) and phospho-eIF2α (normalized to eIF2α) from n = 3 independent replicate cell culture experiments like those shown in Figure 7A-D.

Figure S6. Dose-response of mTOR inhibitors on proinsulin levels. (A) Prior to the start of the experiment, INS1E cells cultured in RPMI-1640 medium were fed every 24 h with a limiting volume of complete RPMI medium containing 11.1 mM glucose (90 µL per cm² surface area), until 48 h. INS1E were lysed at baseline (0 h) or after 4 h incubation in fresh complete RPMI medium (100% amino-acids) in the presence of the indicated concentrations of Torin1. **(B)** Cells were treated identically to those in panel A, except that rapamycin was used at the indicated concentrations. The cells were lysed and resolved by reducing SDS-PAGE and analyzed by immunoblotting with antibodies against phospho-S6, phospho-4E-BP1 and proinsulin (HSP90 is a loading control; molecular weight markers in kDa are indicated at *left*).

Figure S7. Proinsulin levels are regulated by amino-acid availability in pancreatic β-cells. The experimental schema was similar to that shown in Fig. 2D. Cells were fasted by culture with a limiting volume of RPMI, then "preincubated" for 4h in a limiting volume of fresh medium, and then re-fed for an additional 5 h with media containing graded dilutions of RPMI amino-acids (0%, 25%, or 100%) — all at 11.1 mM glucose and with all other components of RPMI held constant. **(A)** Immunoblot analysis of the indicated proteins under all of the conditions described above; Vinculin is the loading control;

molecular weight markers (kDa) are indicated. **(B-F)** Quantitative densitometry from n = 4 independent replicate cell culture experiments (in panel C phospho-eIF2α is normalized to the loading control and in panel D phospho-eIF2α is normalized total eIF2α).

Figure S8. β-cells export selected amino-acids from the cells to the extracellular environment. **(A)** Min6 (mouse-derived) β-cells were fed complete DMEM medium (25 mM glucose) and media collected after 1 or 8 h as described in [29]. Amino-acid concentrations in the collected media were quantified by LC–MS as described in [29]. From five independent wells, replicates were collected at both time points, and the concentration for each amino-acid is shown in the first set of bars (1 h) and the second set of bars (8 h), demonstrating an increase of amino-acid concentration in the media. **(B)** INS1E (rat-derived) β-cells were subjected to the fasting/re-feeding protocol shown in the schema above and incubated in diluted RPMI medium (25% amino-acid content at 11.1 mM glucose) for 5 h. The fold-increase of concentration of each amino-acid in the media from the start (duplicates, right out of the stock bottle) and end of the 5 h incubation (n = 4 independent replicate wells).

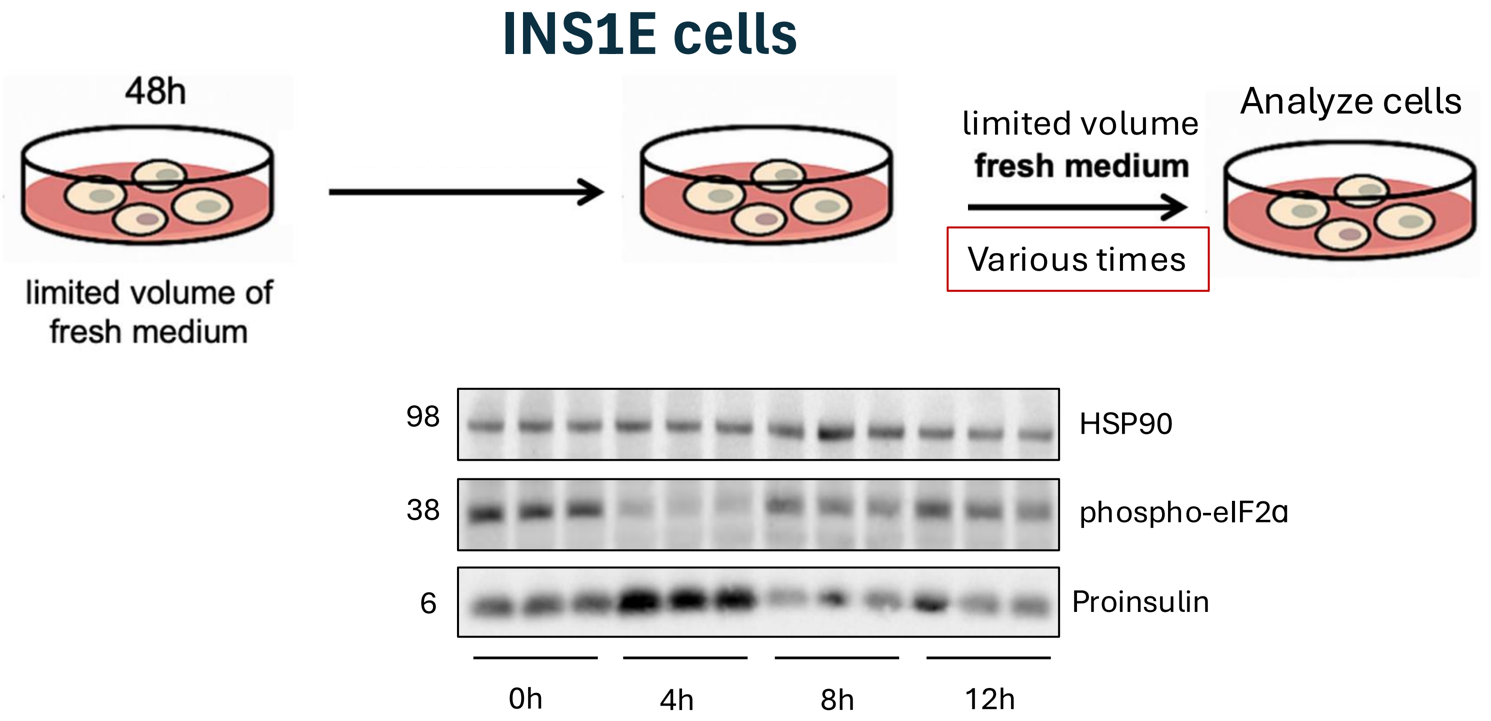

Ruan, Gleason et al., Supplemental Fig. S1

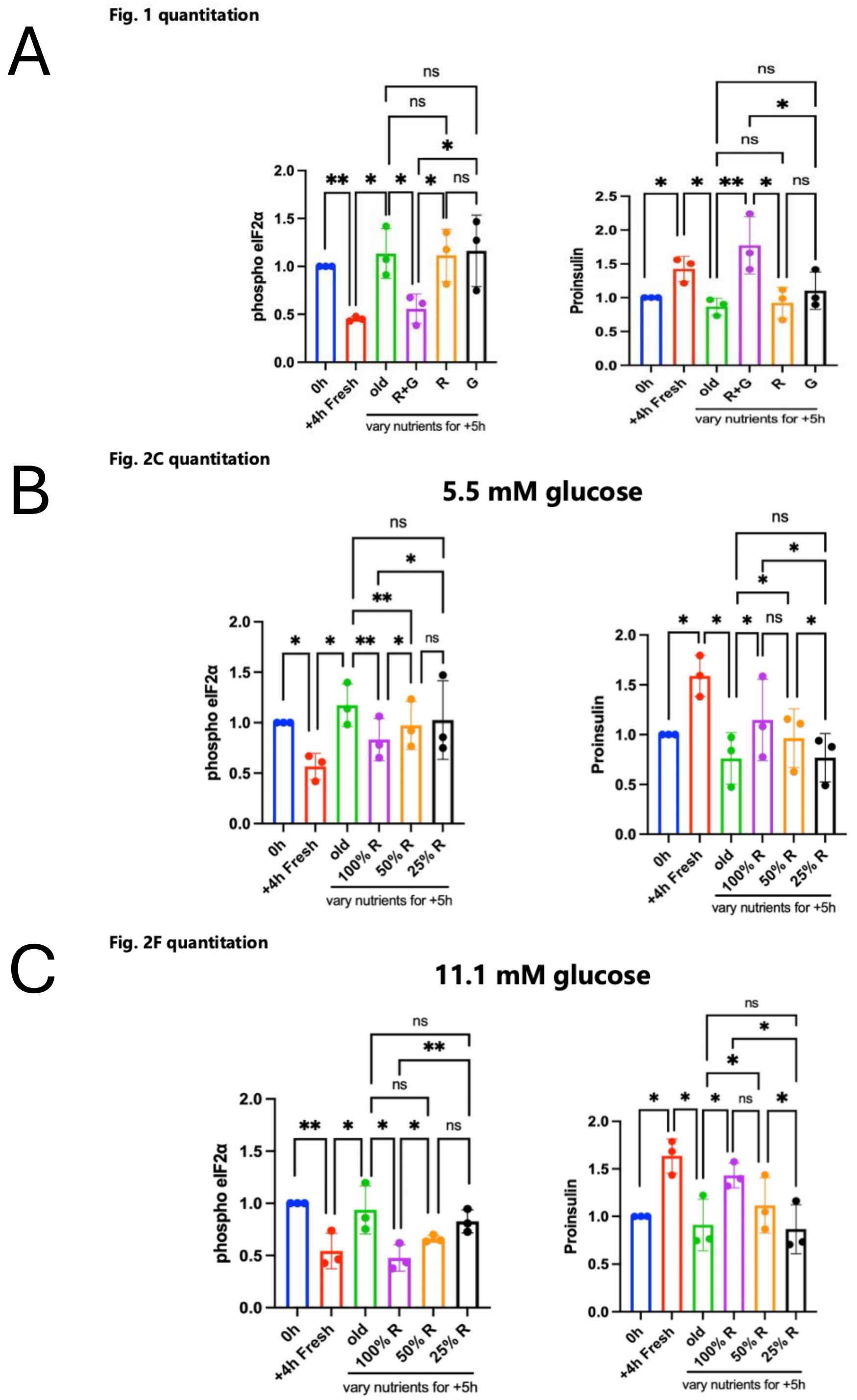

Ruan, Gleason, et al., Supplemental Fig. S2

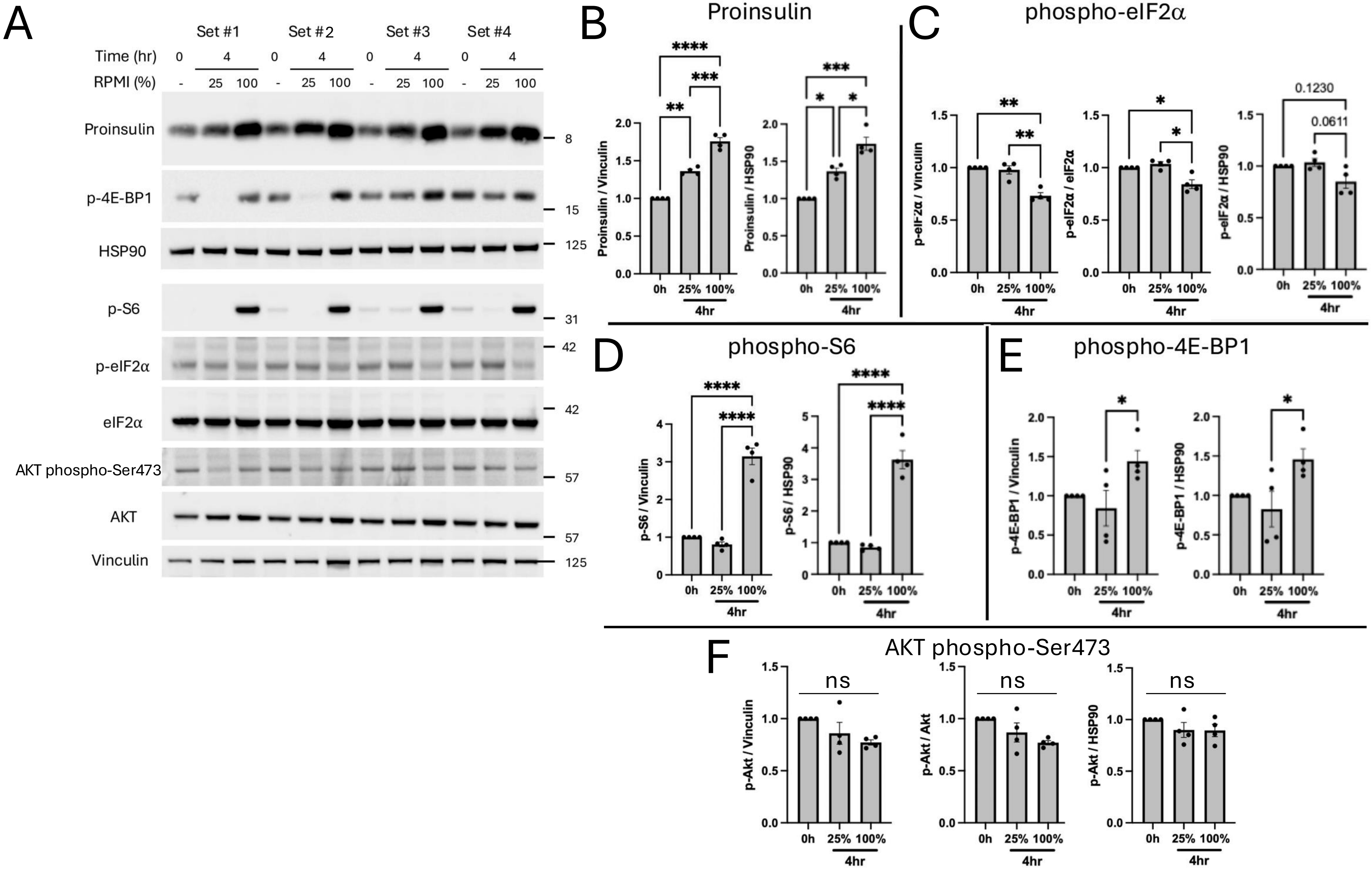

Ruan, Gleason et al., Supplemental Fig. S3

INS1E Total Protein Synthesis

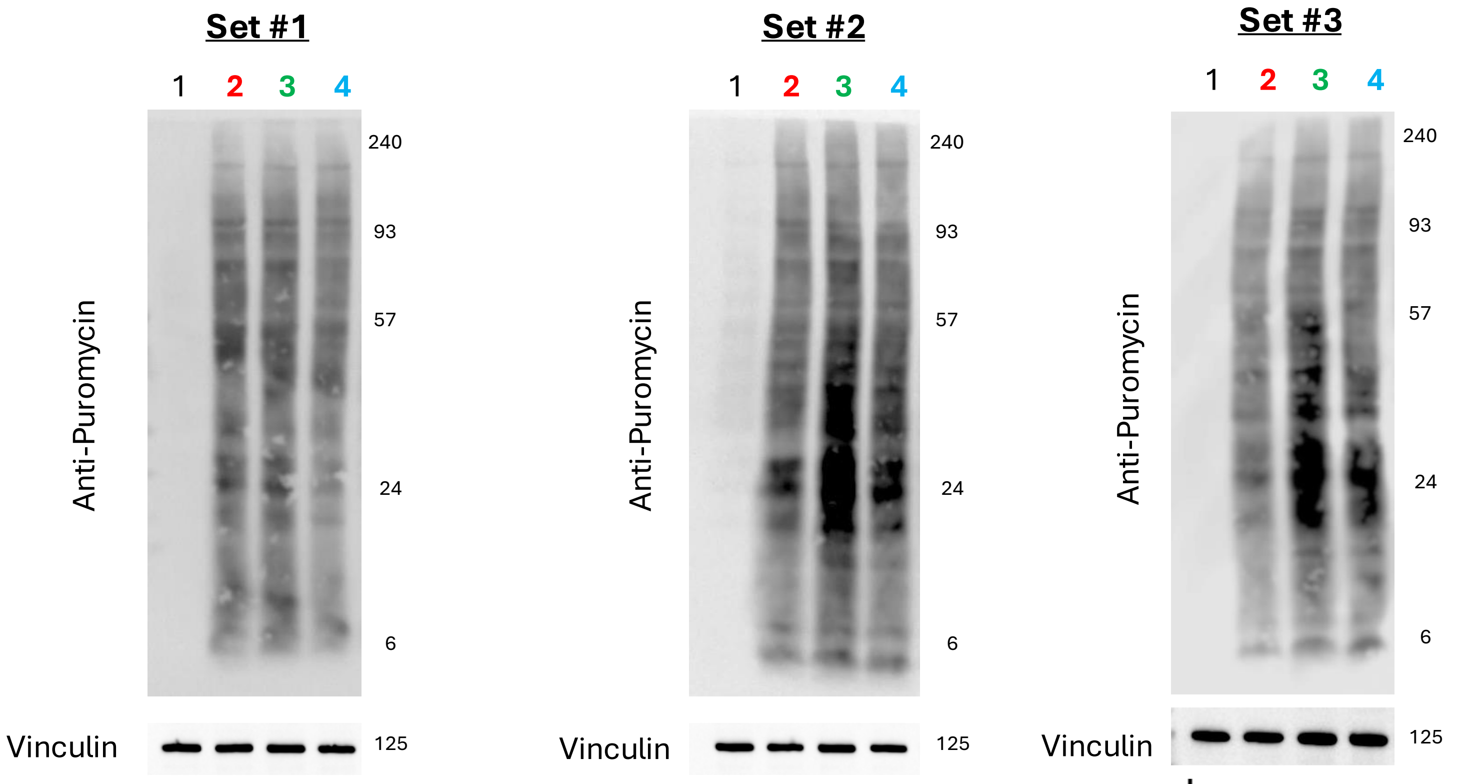

Lane	Condition
1	4h 100% RPMI Control (no puromycin)
2	**4h 25% RPMI (puromycin final 10 min)**
3	**4h 100% RPMI (puromycin final 10 min)**
4	**4h 100% RPMI + Torin1 (puromycin final 10 min)**

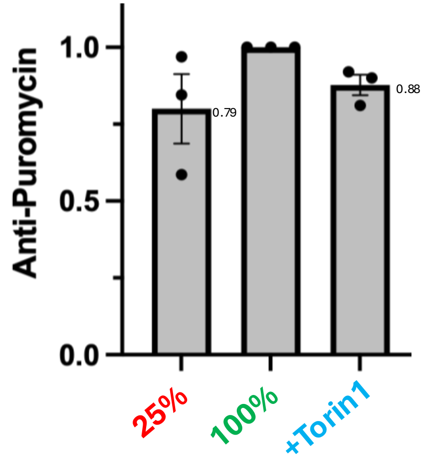

Ruan, Gleason, et al., Supplemental Fig. S4

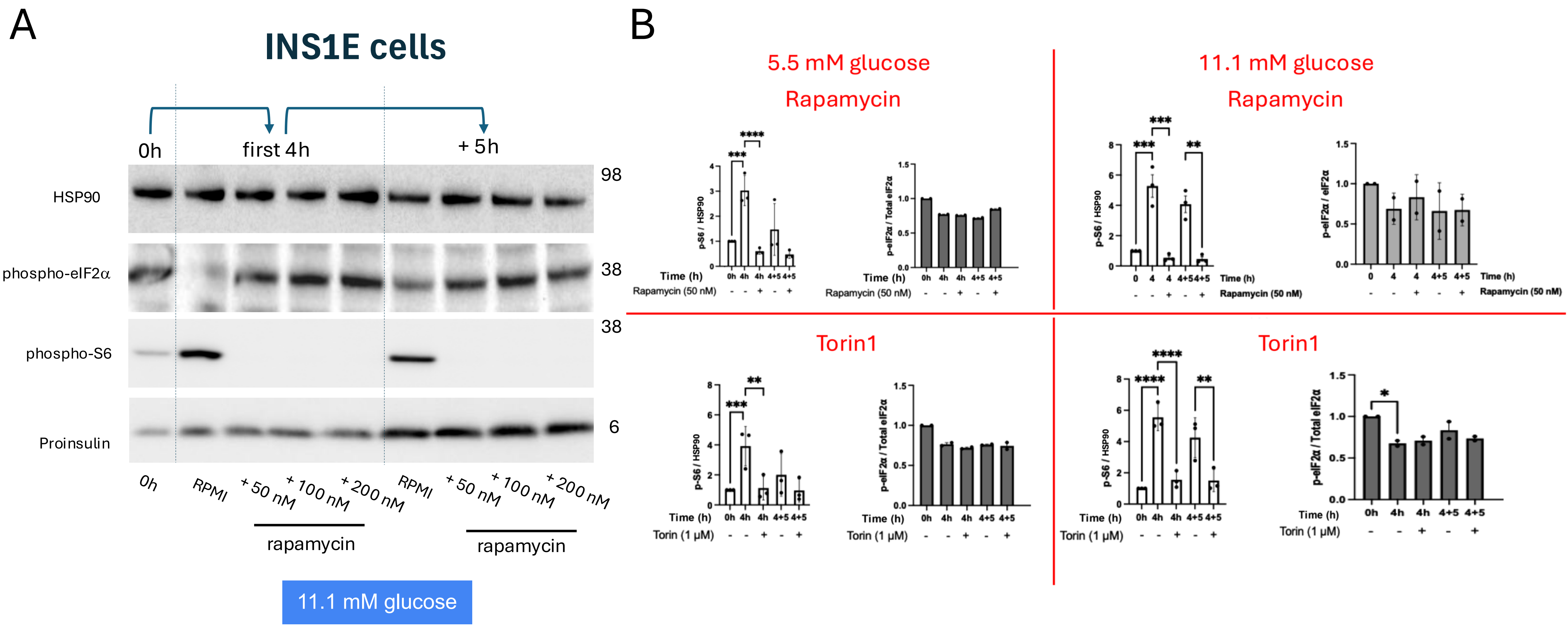

Ruan, Gleason, et al., Supplemental Fig. S5

INS1E cells

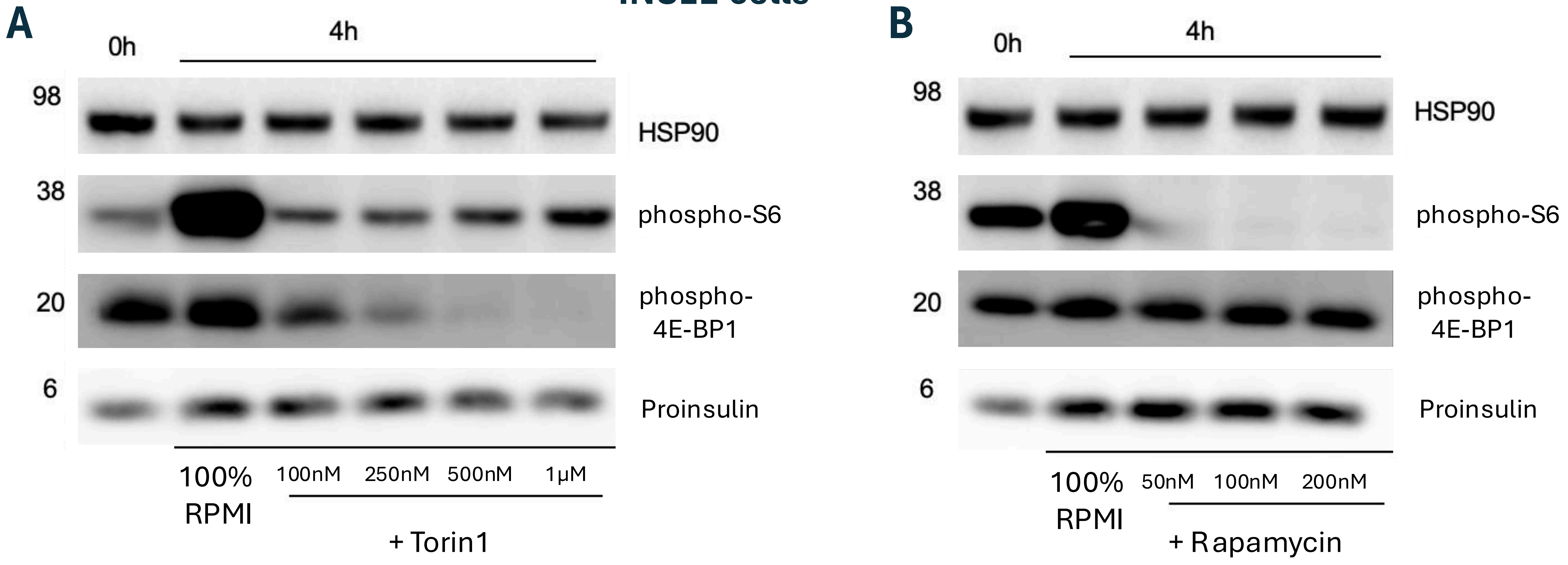

Ruan, Gleason, et al., Supplemental Fig. S6

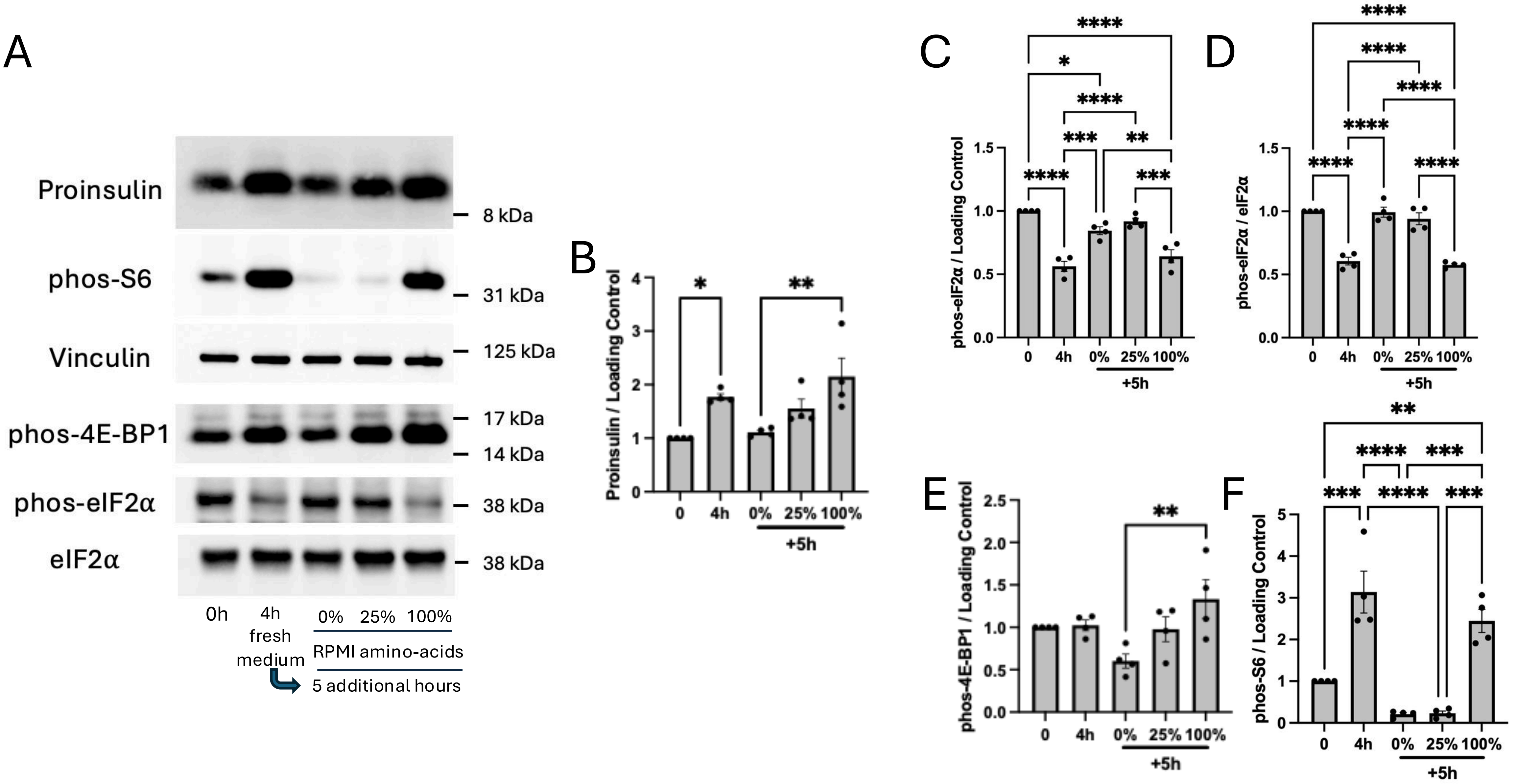

Ruan, Gleason, et al., Supplemental Fig. S7

A

Media bathing Min6 β-cells

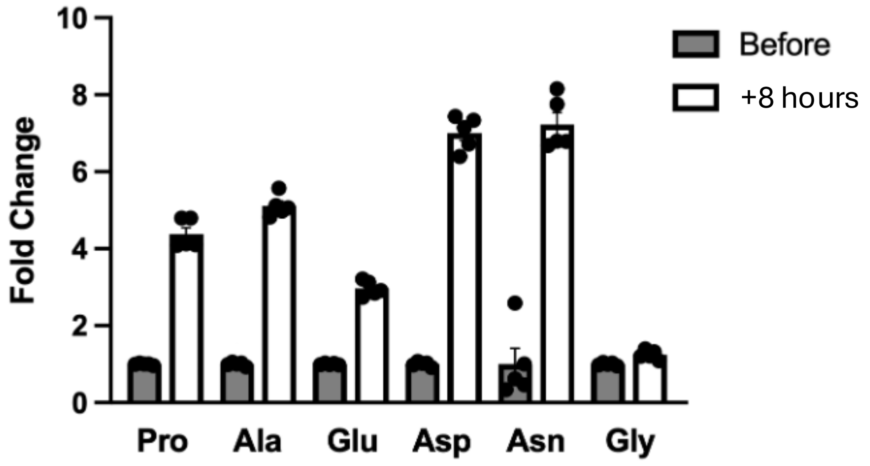

B

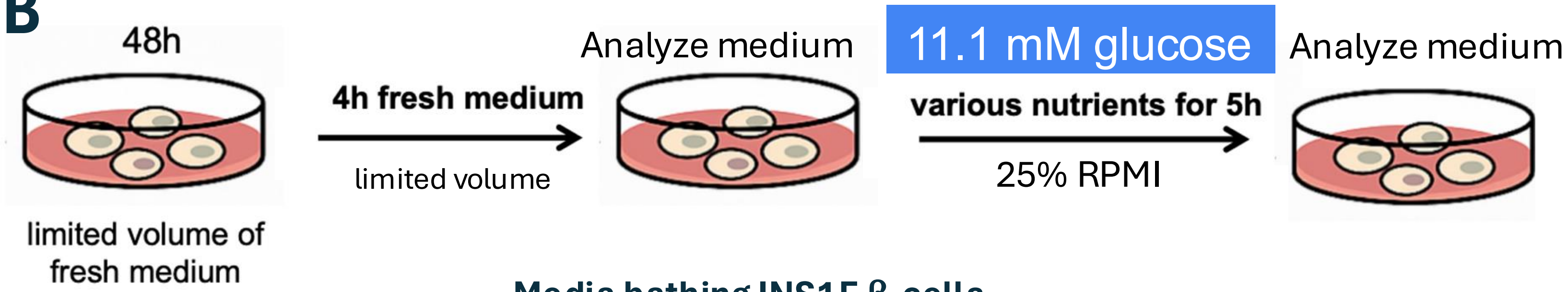

Media bathing INS1E β-cells

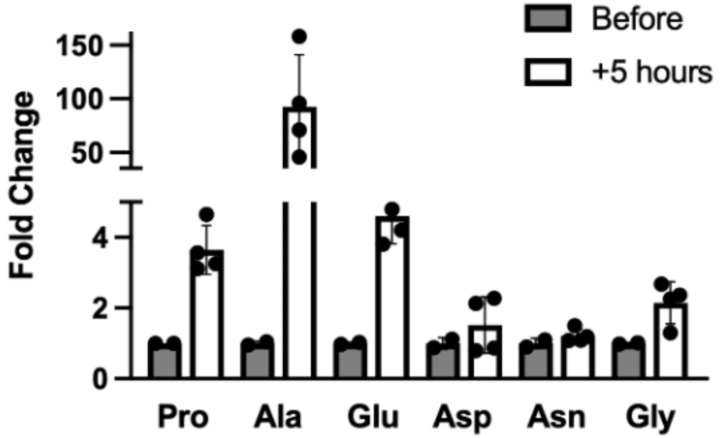

Ruan, Gleason, et al., Supplemental Fig. S8
